# A novel informatics concept for high-throughput shotgun lipidomics based on the molecular fragmentation query language

**DOI:** 10.1186/gb-2011-12-1-r8

**Published:** 2011-01-19

**Authors:** Ronny Herzog, Dominik Schwudke, Kai Schuhmann, Julio L Sampaio, Stefan R Bornstein, Michael Schroeder, Andrej Shevchenko

**Affiliations:** 1 Max Planck Institute of Molecular Cell Biology and Genetics, Pfotenhauerstrasse 108, 01307 Dresden, Germany; 2 Department of Internal Medicine III, Carl Gustav Carus Clinics of Dresden University of Technology, Fetscherstrasse 74, 01307 Dresden, Germany; 3 National Centre for Biological Sciences, Tata Institute of Fundamental Research, GKVK, Bellary Road, Bangalore 560065, India; 4 Biotechnology Centre, Dresden University of Technology, Tatzberg 47-51, 01307 Dresden, Germany

## Abstract

Shotgun lipidome profiling relies on direct mass spectrometric analysis of total lipid extracts from cells, tissues or organisms and is a powerful tool to elucidate the molecular composition of lipidomes. We present a novel informatics concept of the molecular fragmentation query language implemented within the LipidXplorer open source software kit that supports accurate quantification of individual species of any ionizable lipid class in shotgun spectra acquired on any mass spectrometry platform.

## Background

Lipidomics, an emerging scientific discipline, aims at the quantitative molecular characterization of the full lipid complement of cells, tissues or whole organisms (reviewed in [1-4]). Eukaryotic lipidomes comprise over a hundred lipid classes, each of which is represented by a large number of individual yet structurally related molecules. According to different estimates, a eukaryotic lipidome might contain from 9,000 to 100,000 individual molecular lipid species in total [2,5]. Due to the enormous compositional complexity and diversity of physicochemical properties of individual lipid molecules, lipidomic analyses rely heavily on mass spectrometry. A shotgun lipidomics methodology implies that total lipid extracts from cells or tissues are directly infused into a tandem mass spectrometer and the identification of individual species relies on their accurately determined masses and/or MS/MS spectra acquired from corresponding precursor ions [6-8].

The apparent technical simplicity of shotgun lipidomics is appealing; indeed, molecular species from many lipid classes are determined in parallel in a single analysis with no chromatographic separation required. Species quantification is simplified because in direct infusion experiments the composition of electrosprayed analytes does not change over time. Adjusting the solvent composition (organic phase content, basic or acidic pH, buffer concentration) and ionization conditions (polarity mode, declustering energy, interface temperature, etc.) enhances the detection sensitivity by several orders of magnitude [8,9]. In shotgun tandem mass spectrometry (MS/MS) analysis, all detectable precursors (or, alternatively, all plausible precursors from a pre-defined inclusion list) could be fragmented [10]. Given enough time, the shotgun analysis would ultimately produce a comprehensive dataset of MS and MS/MS spectra comprising all fragment ions obtained from all ionizable lipid precursors.

While methods of acquiring shotgun mass spectra have been established, a major bottleneck exists in the accurate interpretation of spectra, despite the fact that several programs (LipidQA [11], LIMSA [12], FAAT [13], LipID [14], LipidSearch [15], LipidProfiler (now marketed as LipidView) [16], LipidInspector [10]) - have been developed for this. Although these programs utilize different algorithms for identifying lipids, they share a few common drawbacks. First, relying on a database of reference MS/MS spectra is usually counterproductive because many lipid precursor ions are isobaric and in shotgun experiments their collision-induced dissociation yields mixed populations of fragment ions. Second, lipid fragmentation pathways strongly depend both on the type of tandem mass spectrometer used (reviewed in [17]) and the experiment settings; therefore, compiling a single generic reference spectra library is often impossible and always impractical. Third, software is typically optimized towards supporting a certain instrumentation platform, while mass spectrometers deliver different mass resolution and mass accuracy and therefore different spectra interpretation algorithms are required. Fourth, the programs offer little support to lipidomics screens, which require batch processing of thousands of MS and MS/MS spectra, including multiple replicated analyses of the same samples.

Therefore, there is an urgent need to develop algorithms and software supporting consistent cross-platform interpretation of shotgun lipidomics datasets [18]. We reasoned that such software could rely upon three simple rationales. First, MS and MS/MS spectra should not be interpreted individually; instead, the entire pool of acquired spectra should be organized into a single database-like structure that is probed according to user-defined reproducibility, mass resolution and mass accuracy criteria. Second, MS/MS spectra should be examined *de novo *in a user-defined way so that adding new interpretation routines (like, probing for another lipid class) should not require modifying the dataset or altering the program engine. Third, it should be possible to apply multiple parallel interpretation routines and, whenever required, bundle them with boolean operations to enhance the analysis specificity.

Here we report on LipidXplorer, a full featured software kit designed in consideration of these assumptions. It relies upon a flat file database (MasterScan) that organizes the spectra dataset acquired in the entire lipidomics experiment. To identify and quantify lipids, the MasterScan is then probed via queries written in the molecular fragmentation query language (MFQL), which supports any lipid identification routine in an intuitive, transparent and user-friendly manner independently of the instrumentation platform.

## Results and discussion

### Shotgun lipidomic experiments: terms and definitions

**Figure 1:**
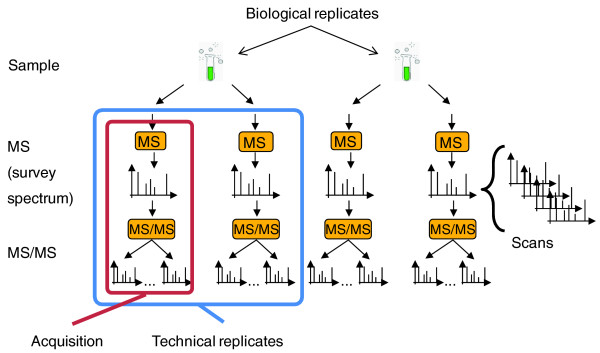
**Making a shotgun lipidomics dataset**. Experiments are repeated in several independent biological replicates for each studied phenotype. Each biological replicate is split into several samples from which lipids are extracted and extracts are independently analyzed by MS. Spectra acquired from the total lipid extract survey molecular ions of lipid precursors, which are subsequently fragmented in MS/MS experiments, yielding MS/MS spectra. Each spectrum is acquired in several scans that are subsequently averaged. A set of MS and MS/MS spectra is termed as an 'acquisition' and several acquisitions are performed continuously making a 'technical replicate'.

Each biological experiment is performed in parallel in several independent replicates. To determine the lipidome in each of these experiments, each biological replicate is split into several samples that are processed and analyzed independently. Total lipid extracts obtained from each sample are infused into a tandem mass spectrometer a few times and several technical replicates are acquired, each providing a full set of MS and MS/MS spectra further termed as an acquisition. Therefore, a typical shotgun experiment yields several hundreds of MS and MS/MS spectra (Figure 1), although many spectra might be redundant because they are acquired in replicated analyses.

During shotgun analyses, spectra are acquired in the following way: within a certain period of time (for example, 30 s) a mass spectrometer repeatedly acquires individual spectra in much shorter intervals (for example, 1 s) that are termed as scans. Subsequent averaging of all related scans into a single representative spectrum increases mass accuracy and improves ion statistics.

Acquisition typically proceeds in a data-dependent mode: first, a survey (MS) spectrum is acquired to determine *m/z *and abundances of precursor ions. Then, MS/MS spectra are acquired from several automatically selected precursors and then the acquisition cycle (MS spectrum followed by a few MS/MS spectra) is repeated. Each acquisition comprises a large number of MS survey spectra and MS/MS spectra from selected precursors, while each spectrum is saved as several individual scans (Figure 1).

A typical lipidomics study might encompass 10 to 100 individual samples, from each of which 10 to 100 MS and 100 to 1,000 MS/MS spectra are acquired. Peaks in MS and MS/MS spectra share three common attributes: mass accuracy (expressed in Da or parts-per-million (ppm)), mass resolution (full peak width at half maximum (FWHM)) and peak occupancy. The two former attributes are determined by mass spectrometer type and equally apply to all peaks detected within the experiment. Contrarily, peak occupancy depends on both instrument performance and individual features of analyzed samples. Even multiple repetitive acquisitions do not fully compensate for under-sampling of low abundant precursors, especially if detected with poor signal-to-noise ratio. Since data-dependent acquisition of MS/MS spectra is biased towards fragmenting more abundant precursors, low abundant precursors might not necessarily be fragmented in all acquisitions. Therefore, the peak occupancy attribute, here defined as a frequency with which a particular peak is encountered in individual acquisitions within the full series of experiments, helps to balance coverage and reproducibility of lipid peak detection.

### Concept and rationale

To support large scale shotgun lipidomics analyses, the software design should address three major conceptual problems: first, the software should utilize spectra acquired on any tandem mass spectrometer; second, it should identify and quantify species from any lipid class that were detected during mass spectrometric analysis; third, it should handle large datasets composed of highly redundant MS and MS/MS spectra, with several technical and biological replicates acquired from each analyzed sample, as well from multiple blanks and controls.

**Figure 2:**
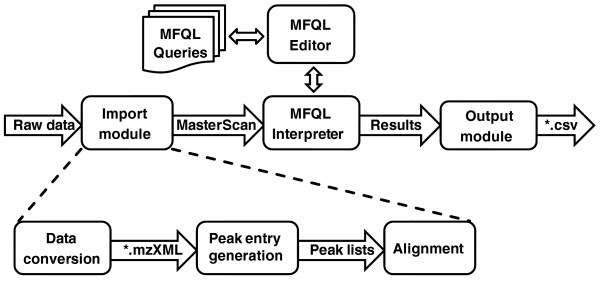
**Architecture of LipidXplorer**. Boxes represent functional modules and arrows represent data flow between the modules. The import module converts technical replicates (collections of MS and MS/MS spectra) into a flat file database termed the MasterScan (.sc). Then the interpretation module probes the MasterScan with interpretation queries written in molecular fragmentation query language (MFQL). Finally, the output module exports the findings in a user-defined format. All LipidXplorer settings (irrespective of what particular module they apply to) are controlled via a single graphical user interface.

To this end, we propose a novel conceptual design that relies upon two-step data processing (Figure 2). First, a full pool of acquired MS and MS/MS spectra is organized into a single flat-file database termed as MasterScan. While building the MasterScan, the software recognizes related MS and MS/MS spectra and aligns them considering the peak attributes. Therefore, there is no need to interpret each spectrum individually, although important features of individual spectra are preserved. The second conceptually novel element is the molecular fragmentation query language, MFQL. We proposed that lipid identification should not rely on the comparison of experimental and reference spectra - whether the latter were produced *in silico *or in a separate experiment with reference substances. Instead, the known or assumed lipid fragmentation pathways can be formalized in a query, which subsequently probes the MasterScan. Spectra interpretation rules are not fixed and are not encoded into the software engine: at any time, users can define new rules or modify the existing rules and apply any number of interpretation rules in parallel.

What are the major conceptual advantages of this design? First, a combination of MasterScan and MFQL enables the interpretation of any MS shotgun dataset acquired on any instrumentation platform and can target any detectable species of any lipid class. Second, aligning multiple related spectra simplifies and speeds up lipid identification in high-throughput screens, improves ion statistics and limits the rate of false positive assignments. To the best of our knowledge, comparable flexibility and accuracy have not been achieved by any available lipidomics software (Table 1).

**Table 1:** Common features of shotgun lipidomics software

**Feature**^**a**^	LipidQA	LIMSA	FAAT	LipID	LipidProfiler	LipidMaps	LipidSearch	LipidXplorer
MS		Yes	Yes	Yes	Yes	Yes	Yes	Yes
MS + MS/MS	Yes				Yes		Yes	Yes
Database of lipid masses		Yes	Yes	Yes	Yes	Yes	Yes	
Database of spectra	Yes							
Database expandability		Yes	Yes	Yes	Yes			Yes
Isotopic correction	Yes	Yes			Yes			Yes
Cross-platform	Yes	Yes	Yes	Yes		Yes	Yes	Yes
Spectra alignment			Yes					Yes
Grouping					Yes			Yes
Batch mode	Yes				Yes			Yes
Offset correction of masses								Yes

^a^List of features: MS, lipid identification solely based on matching precursor masses observed in MS spectra; MS + MS/MS, lipid identification based on MS and MS/MS spectra - required for identifying individual molecular species; Database of lipid masses, lipid identification relies upon a list of expected precursors masses; Database of spectra, lipid identification relies upon a library of reference spectra; Database expandability, users may expand reference databases at will; Isotopic correction, overlapping isotopic clusters are detected and the intensities of corresponding monoisotopic peaks are adjusted; Cross-platform, can process spectra acquired on mass spectrometers from different vendors; Spectra alignment, supports alignment of multiple spectra within the series of experiments; Grouping, supports grouping of spectra within biological and technical replicates acquired from the same sample; Batch mode, supports processing of multiple spectra submitted as a batch; Offset correction of masses, supports adjustment of precursors masses using reference peaks in the MS spectrum.

All programs support direct lipid identification by MS and some also by MS/MS. Most of the software (excepting LipidXplorer) relies upon pre-compiled databases of expected precursor masses or libraries of MS/MS spectra that are either acquired in direct experiments or computed *in silico*. These databases are, in principle, expandable, yet users might not be able to add in new (or putative) lipid classes at will. The identification algorithms are tuned to expected patterns of fragment ions and mass resolution typical for a certain instrument and cross-platform interpretation of spectra is therefore difficult.

The conceptual difference between LipidXplorer and other lipidomics software (Table 1) is that it is fully database-independent. Effectively, each spectra dataset is interpreted *de novo*, while the interpretation rules formalized as MFQL queries may be altered at any time at the user's discretion. Also, LipidXplorer identifications proceed within a pre-processed dataset (MasterScan), which offers the means to adjust processing settings according to the peak attributes. Within the same framework LipidXplorer can accurately interpret spectra acquired on both high- and low-resolution tandem mass spectrometers from different vendors.

LipidXplorer was designed to support a pipeline of lipidomics experiments rather than to assist in identifying lipids in the collection of spectra from a single acquisition. It enables batch processing of all acquisitions made within the series of biological experiments. Users can group individual acquisitions (technical or biological replicates, controls, blanks, and so on) and then compare groups without altering the MasterScan file. Several features were specifically designed to improve the confidence and accuracy of lipid identification and quantification. LipidXplorer improves the mass accuracy by adjusting the masses using offsets to reference peaks. Built-in isotopic correction improves the quantification accuracy by adjusting the abundances of peaks within partially overlapping isotopic clusters.

LipidXplorer outputs the identified lipid species and abundances of user-defined reporter ions in each analyzed sample. We intentionally refrained from programming a module that would recalculate ion abundances into lipid concentrations because quantification routines applied in lipidomics are diverse and strongly project-dependent: they might rely upon several normalization factors (for example, total phosphate content, total protein content, relative normalization to another lipid class, to mention only a few) and employ a palette of internal standards. In high-throughput screens, intensities of precursor ions are directly output into the multivariate analysis software, bypassing the calculation of species abundances (reviewed in [5,19]). At the same time, calculating the concentrations of individual lipids is a simple operation [20] that seldom fails once the accurate basis data (identified lipid species and intensities of reporter peaks) are provided.

The LipidXplorer software is organized in several functional modules (Figure 2) that are controlled by a simple intuitive graphical user interface (GUI; Additional file 1). LipidXplorer starts importing raw mass spectra by averaging individual scans into representative MS and MS/MS spectra. These spectra are further aligned by *m/z *of precursor and fragment ions, respectively, and then MS/MS spectra are associated with the corresponding precursor masses. Spectra-importing routines are instrument-dependent and consider common peak attributes: mass resolution and its change over the full range of *m/z*; minimum peak intensity thresholds specified separately for MS and MS/MS spectra; width of precursor isolation window in MS/MS experiments and the polarity mode. LipidXplorer also corrects observed masses by linear approximation of the mass shift calculated from a few reference masses (if any are detectable in the spectrum). It also pre-filters spectra by user-defined peak intensity and occupation thresholds that are also specified separately for MS and MS/MS modes.

### Scan averaging algorithm

**Figure 3:**
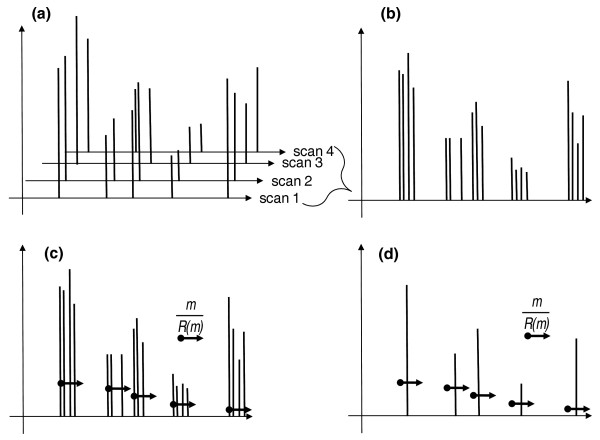
**Scan averaging algorithm**. **(a) **Related individual scans (here as an example we only show four scans) imported as a complete *.mzXML file are recognized. **(b) **Peaks are combined into a single peak list and sorted. **(c) **The full mass range is divided into bins of [*m*; *m*+$\frac{m}{R(m)}$] size, starting from the lowest reported mass. The bold dots stand for the lowest mass of each bin, while the arrow length reflects the bin size $\frac{m}{R(m)}$. Within each bin, masses are weight averaged by peak intensities and stored. The procedure (steps (c) and (d)) is repeated two more times on the binned spectrum (not shown). **(d) **In this way, a single representative average spectrum (d) is produced from several individual scans (a).

While acquiring mass spectra, *m/z *and intensities of peaks might slightly vary within each scan (further, solely for presentation clarity, we will use the mass of a precursor ion *m *instead of its *m/z*). Therefore, averaging individual scans into a single representative spectrum improves the ion statistics and, hence, the accuracy of both measured masses and abundances of corresponding peaks and is commonly applied in proteomics [21,22]. Here we describe a simple linear time algorithm for aligning MS and MS/MS spectra of small molecules (particularly lipids) acquired in large series of shotgun experiments. It assumes that masses pertinent to the same peak are Gaussian distributed within individual scans. The algorithm recognizes related peaks in each individual scan and averages their masses and intensities (Additional file 2). First, the algorithm considers all pertinent scans within the acquisition and combines all reported masses into a single peak list (Figure 3). This list is then sorted by masses in ascending order and averaging proceeds in steps, starting from the lowest detected mass. In every step the algorithm considers mass *m *and checks whether other masses fall into a bin of [*m*; *m*+ $\frac{m}{R(m)}$] width, where *R*(*m*) is the mass resolution at the mass *m*. *R*(*m*) is assumed to change linearly within the full mass range; its slope (mass resolution gradient) and intercept (resolution at the lowest mass of the full mass range) are instrument-dependent features pre-calculated by the user from some reference spectra. All masses within the bin are average weighted by peak intensities according to Equation 1:

(1) $$m_{avg}=\frac{\sum_{m_{i\in B}}\frac{I(m_{i})}{I_{\max}}m_{i}}{\sum_{m_{i\in B}}\frac{I(m_{i})}{I_{\max}}}$$

where *I*(*m*_*i*_) is the intensity of the peak having mass *m*_*i*_, *I*_*max *_is the intensity of the most abundant peak within the bin *B *and *m*_*avg *_is the intensity weighted average mass.

The average mass is then stored as a single representative mass for this bin and the procedure is repeated for the next mass bin. We assume that the variation of peak masses is normally distributed within the bin and therefore the procedure should be repeated several times (Additional file 3). Computational tests (data not shown) suggested that three successive iterations should suffice for complete separation of bins such that masses are collected correctly into their dedicated bins and that no two adjacent bins are closer than the value of $\frac{m}{R(m)}$. One known limitation of this algorithm is that abundant chemical noise might impact binning accuracy. Therefore, we always set the threshold for signal-to-noise ratios of peaks at the value of 3.0, which is a commonly accepted estimate for calculating the limit of detection (LOD) of analytical methods.

### MasterScan: a database of shotgun mass spectra

The MasterScan is a flat file database that stores all mass spectra acquired from all analyzed samples, including technical and biological replicates, blanks and controls. While building the MasterScan, individual acquisitions are processed and stored independently, although users could subsequently combine them into arbitrary groups.

The accurate alignment of MS and MS/MS spectra is a key step in interpreting shotgun lipidomics datasets, yet it is a computationally challenging task. Even successive mass spectrometric analyses of the same sample are not fully reproducible and masses of identical precursors and fragments might vary within certain ranges. Abundances of background peaks are affected by spraying conditions and therefore could hardly serve as robust references. At the same time, not all genuine lipid peaks can be aligned - some peaks might only appear in a few samples, while being fully undetectable in others. Also, the available algorithms for aligning mass spectra are not time-linear and are hardly applicable for shotgun datasets that include both MS and MS/MS spectra [23,24].

The LipidXplorer spectra alignment algorithm (Additional file 4) is similar to the scan averaging algorithm; however, peak masses are averaged without weighting and intensities of all peaks are stored in a list. Each bin is represented by the average mass of individual peaks within the bin. This mass is associated with corresponding intensities in individual spectra, in which the aligned peaks were observed. Note that in tandem mass spectrometric experiments precursor ions are typically isolated within a mass window exceeding 1 Da. Depending on the mass resolution in MS spectra and the actual width of the precursor isolation window, multiple precursor masses might be associated with the same MS/MS spectrum.

**Figure 4:**
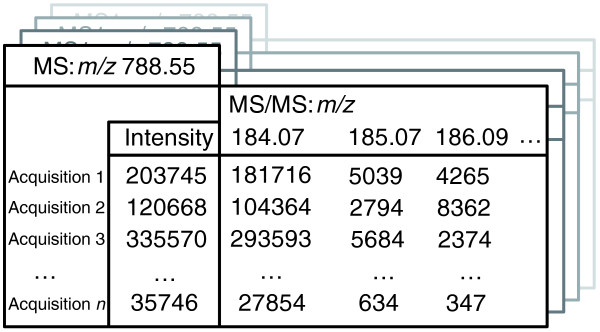
**Organization of a MasterScan file**. LipidXplorer imports and aligns MS and MS/MS spectra into a flat file database MasterScan. It is shown here as a file cabinet addressed at the top-level by precursor masses in the MS spectrum, while their intensities are assigned to individual acquisitions. In this example the lipid precursor with *m/z *788.55 was observed in all acquisitions with an intensity (in arbitrary units) of 203745 in Acquisition 1; 120668 in the Acquisition 2; ... till 35746 in Acquisition *n*. This precursor *m/z *788.55 was fragmented in each acquisition. Masses of fragments were aligned and substituted by the averaged representative masses, while the intensities of corresponding peaks in each individual acquisition were stored. For example, the fragment with *m/z *184.07 has an intensity of 181716 in Acquisition 1; 104364 in Acquisition 2; ..., till 27854 in Acquisition *n*.

Representative masses of all bins, their intensities in individual MS spectra and aligned MS/MS spectra associated with corresponding precursor masses represent the content of a MasterScan file (Figure 4). Effectively, the MasterScan is a comprehensive database for collecting all spectra acquired by shotgun analysis of all samples produced in the full series of biological experiments. The MasterScan reduces data redundancy, compacts the dataset size and increases processing speed because there is no need to probe each individual acquisition successively. In our experience, it usually reduces the total data volume by 45 to 85% because only peak intensities assigned to the representative masses of bins, rather than masses of individual peaks in thousands of original spectra, are stored in the MasterScan.

### The Molecular Fragmentation Query Language (MFQL)

MFQL is the first query language developed for the identification of molecules in complex shotgun spectra datasets. It formalizes the available or assumed knowledge of lipid fragmentation pathways into queries that are used for probing a MasterScan database. Below we introduce its design and present an example of composing a MFQL query for identifying species of phosphatidylcholines lipid class in a typical shotgun dataset.

#### Background and design rationale

**Figure 5:**
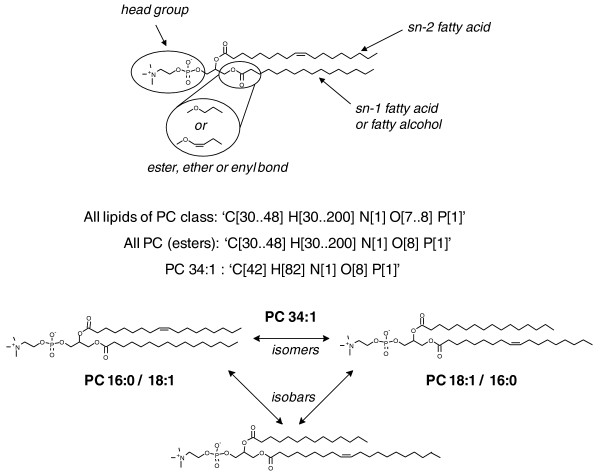
**Structural complexity of lipid species and sum composition constraints**. Let us consider phosphatidylcholines (PC class lipids) as a representative example: PC molecules consist of a posphorylcholine head group attached to the glycerol backbone at the *sn-3 *position, while fatty acid moieties occupy *sn-1 *and *sn-2 *positions (alternatively, a fatty alcohol moiety could be attached at the *sn-1 *position). Fatty acid moieties differ by the number of carbon atoms and double bonds, but also by the relative location at the glycerol backbone, so that isomeric structures having exactly the same fatty acid moieties are possible. Note that isomeric structures are always isobaric, whereas isobaric molecules are not necessarily isomeric. Most generic constraints ('All lipids of PC class' or 'All PC esters') encompass sum compositions of species with all naturally occurring fatty acids. However, because of the fatty acid variability, some species of other lipid classes (such as phosphatidylethanolamines (PE class)) might meet the same constraint. Therefore, for most common glycerophospholipid classes, the characterization of individual molecular species can not rely solely on their intact masses, irrespective of how accurately they were measured. MS/MS experiments that produce structure-specific ions contribute more specific constraints, such as the number of carbons and double bonds in individual moieties, characteristic head group fragment, characteristic loss of a fatty acid moiety, among others. Within a MFQL query, these constraints can be bundled by boolean operations.

MFQL is a specialized query language that is designed for and only usable with a MasterScan database. MFQL queries are search masks for probing lipid spectra for the features stored in the MasterScan, such as precursors and fragment masses and their compositional and abundance relations. Precursors and fragments could be defined directly by their masses, by their chemical sum compositions or by sum composition constraints (sc-constraints; Figure 5).

A typical MFQL query consists of four sections:

DEFINE: defines sum compositions, sc-constraints, masses or groups of masses and associates them with user-defined names.

IDENTIFY: determines where and how the DEFINE content is applied. It usually encompasses searches for precursor and/or fragment ions in MS and MS/MS spectra.

SUCHTHAT: defines optional constraints that are formulated as mathematical expressions and inequalities, numerical values, peak attributes (Additional file 5), sum compositions and functions. Several individual constraints can be bundled by logical operations and applied together.

REPORT: establishes the output format.

A single MFQL query identifies all detectable species of a given lipid class in the dataset, if they share common fragmentation pathways. The MFQL concept takes full advantage of the apparent completeness of shotgun lipidomics datasets that might contain all fragment ions produced from all plausible precursors. In this way MFQL supports parallel application of any shotgun lipidomic approach, such as top-down screening [25,26], multiple precursor and neutral loss scanning [10], multiple reaction monitoring [27,28], among others. The Backus-Naur-Form (BNF) of MFQL is available in Additional file 6.

#### How to compose a MFQL query?

**Figure 6:**
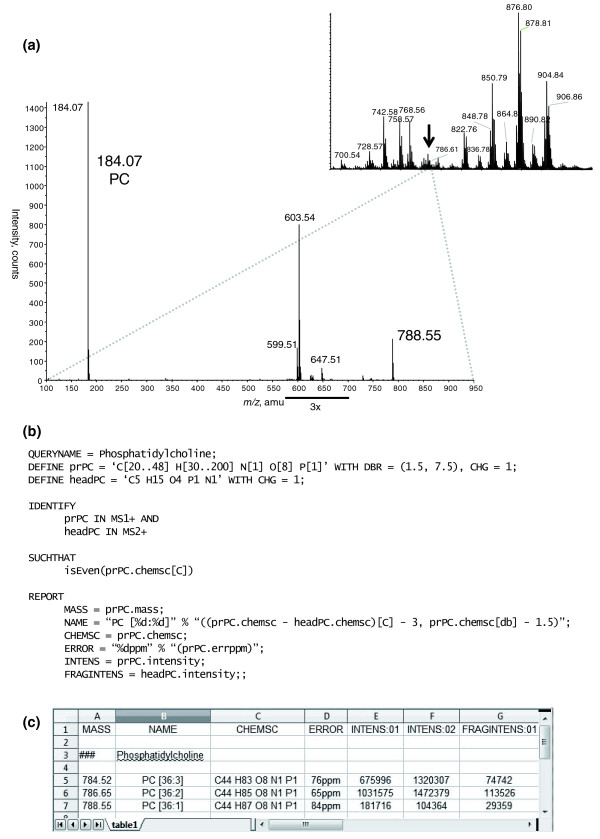
**MFQL identification of phosphatidylcholines (PC)**. The chemical structure of PC is shown in Figure 5. Upon their collisional fragmentation, molecular cations of PC species produce the specific head group fragment with *m/z *184.07 and sum composition 'C5 H15 O4 P1 N1'. **(a) **MS spectrum acquired by direct infusion of a total lipid extract into a QSTAR mass spectrometer (inset). All detectable peaks were subjected to MS/MS. The spectrum acquired from the precursor *m/z *788.55 (designated by arrow) is presented at the lower panel. The precursor ion was isolated within 1 Da mass range and therefore several isobaric lipid precursors were co-isolated for MS/MS and produced abundant fragment ions unrelated to PC. These ions were disregarded by this MFQL query and did not affect PC identification. **(b) **MFQL query identifying PC species, details are provided in the text. **(c) **Screenshot of the output spreadsheet file; column annotation and content is determined by the REPORT section of the above MFQL (see also text for details).

Here we present a MFQL query that formalizes an example scenario for identifying PC species in a shotgun dataset acquired in positive ion mode. In MS/MS experiments, molecular cations of PC produce the specific phosphorylcholine head group fragment having the sum composition of 'C5 H15 O4 N1 P1' and *m/z *184.07. PC species are identification by recognizing this fragment ion in MS/MS spectra and by matching the masses precursor ions in MS to the PC sum composition constraints (Figure 6).

First, let us assign a name to the query:

QUERYNAME = Phosphatidylcholine;

Next, we define the variables used for identifying the species. Our query should identify the singly charged PC head group fragment and therefore:

DEFINE

headPC = 'C5 H15 O4 N1 P1' WITH CHG = +1;

In a shotgun experiment not all fragmented peaks will originate from PCs. For higher search specificity we next define precursors (prPC) that are expected to produce headPC fragment in MS/MS spectra. We impose the sc-constraint on precursor masses: in addition to sum composition requirements, it requests that precursors are singly charged and their degree of unsaturation (expressed as a double bond equivalent) [29] is within a certain range (here from 1.5 to 7.5):

DEFINE

prPC = 'C[30..48]H[30..200]N[1]O[8]P[1]' WITH CHG = +1, DBR = (1.5, 7.5);

Next, the IDENTIFY section specifies that 'prPC' precursors should be identified in MS spectra (termed MS1 in the query) and 'headPC' fragments in MS/MS spectra (termed MS2), both acquired in positive mode. The logical operation AND requests that 'headPC' should only be searched in MS/MS spectra of 'prPC'.

IDENTIFY

prPC IN MS1+ AND

headPC IN MS2+

We further limit the search space by applying optional project-specific compositional constraints formulated in the next SUCHTHAT section. For example, it is generally assumed that mammals do not produce fatty acids having an odd number of carbon atoms. Therefore, we could optionally limit the search space by only considering lipids with even-numbered fatty acid moieties.

SUCHTHAT

isEven(prPC.chemsc[C]);

Here the operator isEven requests that candidate PC precursors should contain an even number of carbon atoms. Since the head group of PC and the glycerol backbone contain 5 and 3 carbon atoms, respectively, this implies that a lipid could not comprise fatty acid moieties with odd and even numbers of carbon atoms at the same time.

By executing the DEFINE, IDENTIFY and SUCHTHAT sections LipidXplorer will recognize spectra pertinent to PC species. The last section REPORT defines how these findings will be reported. This includes annotation of the recognized lipid species, reporting the abundances of characteristic ions for subsequent quantification and reporting additional information pertinent to the analysis, such as masses, mass differences (errors), and so on. LipidXplorer outputs the findings as a *.csv file in which identified species are in rows, while the column content is user-defined. In this example we define five columns, including NAME (to report the species name) and four peak attributes, such as: MASS, species mass; CHEMSC, chemical sum composition; ERROR, difference to the calculated mass; INTENS, intensities of the specified ions reported for each individual acquisition.

REPORT

MASS = prPC.mass;

NAME = "PC [%d:%d]" % "((prPC.chemsc - headPC.chemsc)[C] - 3, prPC.chemsc[db] - 1.5)";

CHEMSC = prPC.chemsc;

ERROR = "%dppm" % "(prPC.errppm)";

INTENS = prPC.intensity;

FRAGINTENS = headPC.intensity;;

It is also possible to define mathematical terms or use certain functions, such as text formatting, on these attributes. The text format implies two strings separated by '%', where the first string contains placeholders and the second string their content. This formatting is used in the NAME string such that the actual annotation convention remains at the user's discretion. In this example two placeholders '%d' of the lipids class name "PC [%d:%d] " are filled with the number of carbon atoms and double bonds in the fatty acid moieties. The number of carbon atoms is calculated by subtracting the sum composition of 'headPC' from the precursor 'prPC' and subtracting 3 for carbons in the glycerol backbone (Figures 5 and 6).

We note that here our assignment of PC species only relied upon their precursor masses and the identification of the specific head group fragment in their MS/MS spectra. Therefore, we could only annotate the species by the total number of carbon atoms and double bonds in both fatty acid moieties (like PC 36:1), but we could not determine what these individual moieties really were.

### Validation of the LipidXplorer algorithms

LipidXplorer has been subjected to extensive validation in two ways. First, we tested scan averaging, spectra alignment and isotopic correction routines in a series of experiments with specifically designed datasets. Second, we benchmarked overall LipidXplorer identification performance against available lipidomics software using the *Escherichia coli *total lipid extract as a sample and the curated list of identified species as a reference.

#### Validation of scan averaging

We compared scan averaging in LipidXplorer with the related procedure implemented in Xcalibur software - a dedicated tool for processing spectra acquired on Thermo Fisher Scientific mass spectrometers and the *de facto *standard in processing of high-resolution spectra. To this end, we acquired a dataset of MS spectra of 325 lipid extracts on a LTQ Orbitrap mass spectrometer with a mass resolution of 100,000. Each acquisition consisted of 19 scans, which were independently averaged by Xcalibur and LipidXplorer. Then, each pair of averaged spectra within the same acquisition was aligned by peak masses, such that the two masses *m*_*1 *_and *m*_*2 *_were considered identical if |*m*_*2 *_- *m*_*1*_| <$\frac{m_{1}}{R(m_{1})}$, where mass resolution *R *= 100,000. To test if the algorithm performance was affected by chemical noise in the aligned spectra, we selected peaks with intensities above 1%, 0.5% and 0.1% of the base peak intensity. It is usually assumed that the typical dynamic range (the ratio of intensities of the most abundant to the least abundant signal) in Orbitrap spectra is less than 1,000-fold [30] and therefore the intensity threshold of 0.1% corresponds to peaks that are at the edge of reliable detection. We found that the averaging algorithm performed well on peaks selected at the lowest threshold: only 7% of peaks mismatched, while mass differences between the aligned peaks were, on average, within 0.3 ppm and their intensities differed by less than 3%. Spearman rank correlation factors (SRCFs) were calculated using the intensities of aligned peaks and the average SRCFs are presented in Table 2. We concluded that the simple algorithm implemented in LipidXplorer performed equally well as the related algorithm in Xcalibur (Additional file 7).

**Table 2:** Comparison of scan averaging algorithms in Xcalibur and LipidXplorer

Intensity threshold	1%	0.5%	0.1%
Number of peaks	158.40 ± 23.57	237.62 ± 37.36	736.22 ± 128.71
Mass difference, ppm	0.06 ± 0.09	0.08 ± 0.09	0.30 ± 0.09
Intensity difference, %	0.61 ± 0.87	0.72 ± 0.86	3.00 ± 1.24
Spearman rank correlation	0.99 ± 0.02	0.98 ± 0.02	0.94 ± 0.03
Mismatched masses, %	1.45 ±1.44	2.37 ± 1.57	7.06 ± 2.36

All values are average ± standard deviation.

#### Validation of isotopic correction

The isotopic correction algorithm adjusts the intensities of peaks within partially overlapping isotopic clusters of neighboring lipid species [7,12,20]. The algorithm computes the expected profiles of isotopic clusters from the sum compositions of identified lipids and corrects corresponding peak intensities in both MS and MS/MS modes.

**Figure 7:**
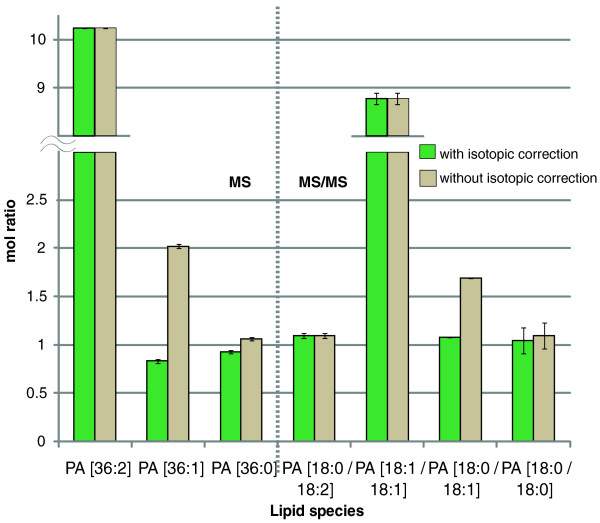
**Validation of the isotopic correction algorithm using a PA mixture**. Molar ratios of PA standards were determined in four replicates with and without isotopic correction of abundances of peaks within partially overlapping isotopic clusters. Molar ratios in MS spectra were determined from the abundances of precursor peaks and in MS/MS spectra as the sum of the abundances of acyl anions of the fatty acids moieties. Error bars stand for standard deviations from the average molar ratios.

To test the algorithm, we injected a mixture of four phosphatidic acid (PA) standards with the molar ratio 1:9:1:1 into a LTQ Orbitrap Velos mass spectrometer and acquired MS and MS/MS spectra. The two standards PA 18:0/18:2 and PA 18:1/18:1 have the same exact masses; therefore, in MS spectrum the ratio of precursor ion intensities of 10:1:1 was anticipated. For species quantification in MS/MS spectra, we summed the intensities of acyl anions of corresponding fatty acid moieties expecting the ratio of 1:9:1:1 (Figure 7).

Measured molar ratios agreed with the expected ratios and ratios calculated from computationally simulated spectra (data not shown). We underscore that isotopic correction is absolutely required to determine the content of relatively low abundant species. Even at the moderate dynamic range of 1:9, the abundance of PA 18:0/18:1 would have been drastically overestimated in both MS and MS/MS measurements (Additional file 8).

#### Validation of the spectra alignment algorithm

The algorithm should recognize related peaks within the submitted spectra and attribute them to mass bins in a resolution-dependent manner, while individual peak abundances should be preserved. An ideal validation test should encompass a large collection of real-life spectra, while in each spectrum the correct (rather than measured) masses of peaks observed even at the lowest signal-to-noise ratio should be exactly known. Since this is unfeasible, we validated the algorithm in two separate tests. In the first test, peak abundances were effectively disregarded, yet the correct masses were exactly known and the dataset composition was controlled. The second test relied on a compendium of real-life spectra of total lipid extracts having typical distribution and variability of abundances of genuine lipid peaks, along with a large number of background peaks and chemical noise. However, the exact composition of lipid species in each sample was not known.

We first designed an experiment in which several spectra were computationally generated from a template spectrum and aligned in a MasterScan. The abundances of peaks were then correlated with the abundances of peaks in the original template spectrum. We designed the template spectrum such that the distance between the two adjacent peaks with the masses *m*_*1 *_and *m*_*2 *_was $\frac{m_{1}}{R(m_{1})}$, where *R *= 500. Within a mass range of 500 to 945, which covers most lipid precursors, the template contained 319 peaks that were spaced, on average, by a distance of 1.4 Da. From this template we generated 256 spectra in which masses of peaks were randomly selected from Gaussian distributions having the centroid *m *and σ = $\frac{2m}{R(m)}$, where *R *= 100,000 and *m *is the corresponding mass from the template spectrum. Note that, under selected resolution and spacing, peaks in the simulated spectra did not overlap.

Conventionally, LipidXplorer successively repeats spectra binning three times. However, for this test only, we configured LipidXplorer such that peaks were binned one, two and three times. After importing the spectra, we anticipated that all 319 peaks of the template spectrum should be present in the MasterScan and that occupation of individual peaks through all 256 spectra should mirror Gaussian distribution, if peaks were only binned once. Therefore, we expected to find 319 peaks with an average occupation of 0.68, since this is the number of peaks falling into the rage of [*m*- σ, *m*+σ] of the distribution, which equals a bin size of $\frac{m}{R(m)}$.

Indeed, we found that after one-step binning 319 peaks were correctly aligned and had an average occupation of 0.65 (Table 3). The average mass difference between the template and aligned peaks were 0.9 mDa. As expected, repeating the procedure substantially improved the binning accuracy (Additional file 9).

**Table 3:** Computational validation of the peak alignment algorithm

Number of binning cycles	Average peak occupation	Average mass difference, ppm
1	0.65 ± 0.05	1.3 ± 0.8
2	0.87 ± 0.08	1.6 ± 0.7
3	0.97 ± 0.04	0.4 ± 0.4

However, this test assumed that in the aligned spectra no unrelated peaks fall into the same mass bin, which is unrealistic in real-life shotgun spectra. Therefore, we next tested if the alignment accuracy was affected by the complexity of the analyzed lipid mixtures and by chemical noise. To this end, we compared lipid species identified by LipidXplorer in individual spectra and in the same spectra aligned within the MasterScan.

**Figure 8:**
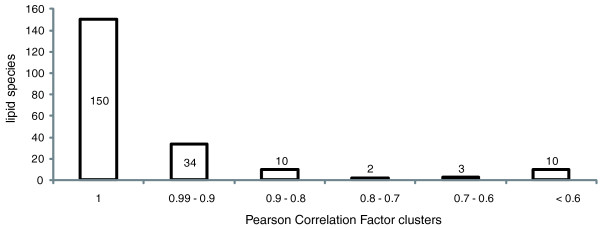
**Pearson correlation factors of peak abundances in the MasterScan and individual spectra**. In total, the dataset consisted of 128 high resolution MS spectra of total lipid extracts in which 219 peaks of individual lipid species were recognized. The exact number of peaks assigned to lipid species is provided for each PCF bin. The average PCF calculated for the entire dataset had a value of 0.94

Using 128 MS spectra of total lipid extracts of different human blood plasma samples [25], we compiled a MasterScan file in which individual spectra were mass-aligned as described above. In parallel, each of these 128 spectra was submitted to LipidXplorer, lipid species were identified under the same settings, and then the spectra were aligned by identified species (not by peak masses, as in the MasterScan). We note that, in both tests, the intensities of peaks in individual spectra were preserved. We then computed Pearson correlation factors (PCFs) between the intensities of peaks of the same lipid species in the same acquisition, either determined in the raw 'as submitted' spectrum (lipids were identified in individual spectra), or aligned within the MasterScan file (lipids were identified by probing the MasterScan). We anticipated that accurate alignment of multiple spectra would increase the mass accuracy of each individual peak and improve peak identifications. A total of 218 lipid species was recognized by both methods. Of these, three and six species were not identified in the MasterScan and in individually processed spectra, respectively. We compared the intensities of lipid peaks identified by both methods by calculating the PCFs of their intensity vectors (Figure 8) and found that the PCFs of 15 lipid species out of the total of 218 fell below 0.8. Case-by-case inspection of these showed that isotopic clusters of three species in individual spectra were altered by background or spray instability. The remaining 12 lipid species were very low abundance and their peak intensities were below 0.1% of the intensities of base peaks in corresponding spectra. We therefore concluded that, while building a MasterScan, mass-alignment of peaks was, in general, correct. The full test dataset is available in Additional file 10.

### Benchmarking the lipid identification performance

We benchmarked the LipidXplorer performance in two ways. First, we provided an estimate of the rate of false positive identifications by shotgun analysis of a total lipid extract. Second, we compared LipidXplorer identification performance with other programs that support shotgun lipidomics experiments by interpreting peak lists produced from MS and MS/MS spectra.

We note that the composition of any complex real-life lipid extract might not be exactly known and it is therefore difficult to judge if any particular identification is a false positive. To circumvent this problem, we first produced a dataset of MS and MS/MS spectra by analyzing a commercially available total lipid extract of *E. coli *on a LTQ Orbitrap XL mass spectrometer using data-dependent acquisition in negative ion mode. It is known that, upon collision-induced dissociation, molecular anions of glycerophospholipids produce abundant acyl anions of their fatty acid moieties that enable unequivocal identification of individual molecular species [31]. The glycerophospholipidome of wild type *E. coli *comprises bulk quantities of phosphatidylethanolamines (PE class) and phosphatidylglycerols (PG class) and minor amounts of PA [32-34] that are identifiable with any available software. Also *E. coli *does not produce lipids with polyunsaturated fatty acid (PUFA) moieties [33,35]. Therefore, we reasoned that species of other glycerophospholipid classes (such as phosphatidylinositols (PI class) and phosphatidylserines (PS class)) or any species containing PUFA, if identified by the software, will likely represent false positives. Cardiolipins, another major component of the *E. coli *lipidome, could be detected as both singly and doubly charged molecular anions, which might lead to inconsistent interpretations of both MS and MS/MS spectra by different software. We therefore deliberately omitted the identification of cardiolipins from our benchmarking protocol.

Lipid composition of the standard *E. coli *extract was determined in two ways. First, a list of species was produced by manual interpretation of spectra acquired on a LTQ Orbitrap XL machine with high mass resolution of 100,000 and 15,000 (FWHM, *m/z *400) in MS and MS/MS modes, respectively, which allowed us to impose stringent constraints for matching of both precursor and fragment peaks. In this way, we identified 38 lipid species of the PE, PG and PA classes. Independently, the same extract was analyzed by the multiple precursor ion scanning (MPIS) method on a quadrupole time-of-flight mass spectrometer [16]. The interpretation of the MPIS dataset by LipidProfiler software confirmed 36 species representing 95% of the species identified manually. The intersection of species identified by manual interpretation of high resolution spectra and by MPIS/LipidProfiler was assumed as a reference list. Within the reference list, 78% of lipids were also present in the LIPIDMAPS database (Table 4). We underscore that, while compiling a reference list, we aimed to provide the most conservative minimalistic estimate of the lipid composition, that is, we included only the species that must be identifiable in any further software tests. This does not imply that PE and PG species other that in the reference list are necessarily false positives.

**Table 4:** Benchmarking LipidXplorer identification performance using the *E. coli *lipidome

Lipid class	Reference list	**LipidMaps**^**a**^	**LipidQA**^**b**^	LipidSearch	LipidXplorer
True positives					
PA^c^	0	0	0/1	0/1	0/0
PE	21	18	12/14	14/21	21/27
PG	15	10	8/13	9/17	15/25
Compliance^d^, %			56	64	100
False positives					
PS			2	0	0
PI			0	0	0
PUFA species^e^			7	2	1
Total			9	2	1

^a^The lipid species database is at [53]. ^b^The number of identified species is presented as 'Number of species that belong to the reference list/Total number of identified species'. The numbers are presented separately for each class. ^c^PA is a very minor (<0.01 mol%) component of the *E. coli *lipidome. ^d^Compliance is a ratio of the total number of identified species that belong to the reference list to the total number of identified species. It is calculated for species of all three lipid classes together. ^e^Putative lipid species with polyunsaturated fatty acids (PUFAs) were searched within PA, PE and PG lipid classes. With respect to PUFAs here, we assumed fatty acids having more than two double bonds to account even for the rarest instances when a moiety might contain one double bond and one cyclopropane ring.

In summary, our software benchmarking procedure relied upon the following rationale: we estimated the rate of false negative identifications by comparing the software output to the reference list and we estimated the rate of false positive identifications by forcing the software to identify species from lipid classes that are not produced by *E. coli*. For the latter test, we only considered the lipid classes whose precursors readily produce molecular anions and whose masses might overlap with precursors of genuine *E. coli *lipids (PE, PG, PA) in low resolution mass spectra. Although LipidXplorer could restrict the search space by sc-constraints and, hence, reduce the expected rate of false positives (data not shown), for better consistency with other tested programs it was set to report hits with fatty acid moieties having up to 22 carbon atoms and up to 6 double bonds.

A separate dataset was acquired in eight technical replicates from the same *E. coli *extract under the low mass resolution of 800 for both MS and MS/MS modes, which is common for triple quadrupole or ion trap instruments. This dataset was independently processed by LipidXplorer, LipidQA and LipidSearch programs (Table 4). LipidQA and LipidSearch could only process each technical replicate independently. Therefore, their output was aligned by the reported lipid species and species identified in less than four (out of the total of eight) replicates were discarded. The same criterion was applied using an occupation threshold of 50% while testing LipidXplorer.

LipidXplorer produced a total of 53 identifications, which included 36 (100%) species from the reference list plus another 17 species (see Additional file 11 for corresponding MFQL queries). According to the above convention, one species was declared a false positive. Both LipidQA and LipidSearch reported fewer species from the reference lists and more false positives (Table 4). A full list of species identified by all software tools is presented in Additional file 12.

Based on these findings, we concluded that LipidXplorer outperformed the currently available software in interpreting shotgun lipidomics datasets.

### Benchmarking LipidXplorer speed

Importing a dataset of 32 samples each consisting of 55 MS and 110 MS/MS scans in *.mzXML format took 59 s on an Intel Core 2 Duo CPU (T9300; 2.50 GHz) computer under Windows Vista. The total size of the *.mzXML files was 45 MB, whereas the size of the produced MasterScan file was only 3.35 MB. LipidXplorer identification of species of six lipid classes (PC, PC-*O *(1-alkyl-2-acylglycerophosphocholines), PE, PE-*O *(1-alkyl-2-acylglycerophosphoethanolamines), SM (sphingomyelins) and TAG (triacylglycerols)) required 59 s.

To test how the processing speed of LipidXplorer is affected by the spectra dataset size, we imported mzXML files totaling 168 MB that comprised 248 MS acquisitions each of approximately 2,400 peaks. Building the MasterScan file took 13 minutes on the same desktop PC and required 0.7 GB of RAM. Subsequent screening of the 29.1 MB MasterScan file with 16 MFQL queries required only 6.5 s. We note that a MasterScan is only built once from all spectra acquired in the project. Further interpretation of the dataset, including repetitive screening for other lipid classes or using alternative signature ions, does not require changing the MasterScan. Although LipidXplorer does not explicitly restrict the size of mzXML files, in our experience a dataset of 500 acquisitions each comprising 2,500 peaks might be a practical limit for desktop computers having up to 4 GB of RAM.

### Enabling functionalities of LipidXplorer

Using MasterScan and MFQL within LipidXplorer software has two important analytical implications. First, LipidXplorer accurately processes MS and MS/MS spectra acquired on different tandem mass spectrometers whose mass resolution varies from the unit (triple quadrupoles, ion traps) to 100,000 (Orbitrap). Second, the software identifies any individual lipid species or entire lipid classes that were ionized and fragmented during the shotgun experiment.

#### LipidXplorer supports mass resolution-dependent interpretation of shotgun mass spectra

Mass resolution and mass accuracy of detected peaks are determined by the type of employed tandem mass spectrometer. LipidXplorer imports spectra in generic mzXML format and converters from proprietary formats to mzXML are available for major instrument platforms. Here we provide evidence that LipidXplorer consistently and accurately interprets spectra acquired at different mass resolution and accuracy.

**Figure 9:**
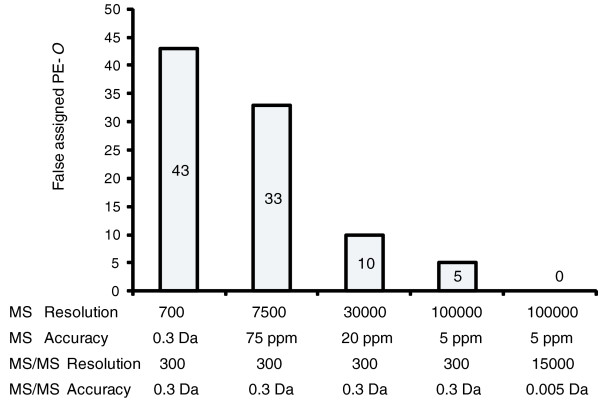
**LipidXplorer accurately interprets both high and low resolution mass spectra**. The number of PE-*O *species falsely assigned by LipidXplorer software in shotgun analysis of a total *E. coli *lipid extract under different target mass resolutions.

We performed several independent shotgun analyses of an *E. coli *total lipid extract on a LTQ Orbitrap XL mass spectrometer under different target mass resolution settings (described as experiments I to V in Materials and methods; Figure 9) and interpreted the datasets with LipidXplorer. Within the series of successive MS experiments, mass accuracy of the Orbitrap analyzer was dependent only on the target resolution *R*; therefore, for matching the masses of lipid species, we assumed that the tolerance at mass *m *equals $\frac{m}{R(m)}$. We were interested in the number of false positive assignments of detected peaks to PE-*O *species that are not produced in *E. coli*, but closely resemble the structure and often have masses isobaric with abundant PE species. The difference in exact masses of isobaric PE and PE-*O *species is 36.4 mDa and their peaks can be distinguished in high resolution spectra [26,27]. Since the same sample was analyzed each time and the same precursor and fragment masses were expected, the experiment provided a consistent dataset for benchmarking LipidXplorer performance in interpreting spectra acquired on low- and high-resolution instruments.

Diacyl (PE) and alkylacyl (PE-*O*) lipids were distinguished by assigning the correct sum compositions to peaks observed at a mass resolution of 30,000. The number of false assignments to PE-*O *dropped from 33 at a MS resolution of 7,500 to 10 at a MS resolution of 30,000, which, as expected, distinguished peaks with a mass offset of approximately 30 mDa. Increasing mass resolution in MS spectra up to 100,000 further decreased the number of false positives, yet did not eliminate them completely. When the mass resolution was also increased in MS/MS mode up to 15,000 and enabled to match fragment masses with an accuracy of better than 5 mDa, the number of false positive assignments dropped to zero (Figure 9). Hence, we demonstrated that LipidXplorer takes full advantage of the high mass resolution and mass accuracy of a hybrid tandem mass spectrometer. It has also become apparent that averaging and alignment of related peaks in multiple experiments did not compensate for the limited identification specificity of low resolution machines (Additional file 13).

#### LipidXplorer supports consistent cross-platform identification of lipids

By its design and operational principles, LipidXplorer is not tethered to any particular mass spectrometry platform. The program imports shotgun spectra as instrument-independent peak lists or mzXML files. When building a MasterScan, LipidXplorer only considers a few generic features of raw MS and MS/MS spectra, such as mass resolution and mass accuracy, while MFQL adapts lipid identification routines to machine-dependent molecular fragmentation pathways. This implies that even if raw spectra are acquired on different machines and using different analytical modes (MS or MS/MS), their LipidXplorer interpretation should result in quantitatively consistent profiles provided the intensities of selected precursor and/or fragment peaks adequately represent the abundances of lipid species. To substantiate this, we validated LipidXplorer cross-platform performance in two steps. First, we demonstrated that lipid quantification by LipidXplorer corroborates an established independent analytical method that relies on a different instrument, operation mode and software; this ensured that LipidXplorer interpretations were correct. Second, we employed LipidXplorer for interpreting shotgun datasets of MS and MS/MS spectra acquired on different instruments and demonstrated that it produced quantitatively concordant molecular species profiles.

To this end, we analyzed a total lipid extract of *E. coli *on the LTQ Orbitrap Velos by MS and data-dependent MS/MS. Then, the same extract was analyzed on a quadrupole time-of-flight mass spectrometer QSTAR Pulsar *i *by MS and MS/MS and also by the MPIS method, which is a unique feature of QSTAR machines [16,31]. The dataset of MPIS spectra was processed using LipidProfiler software. For better consistency, the mass resolution of the Orbitrap was set at 7,500 such that it was close to the mass resolution of the QSTAR. MS and MS/MS spectra were imported into MasterScan databases as mzXML files and the same MFQL queries (Additional file 11) were applied to identify and quantify 24 major species (15 from PE and 9 from PG lipid classes) that were detected in all analyses with good signal-to-noise ratios, which was important for consistent comparison of independent experiments. MS quantification relied on the intensities of intact molecular anions of corresponding species, while for MS/MS quantification the MFQL queries reported the intensities of acyl anion fragments of corresponding fatty acid moieties of each fragmented lipid precursor [10,16].

**Figure 10:**
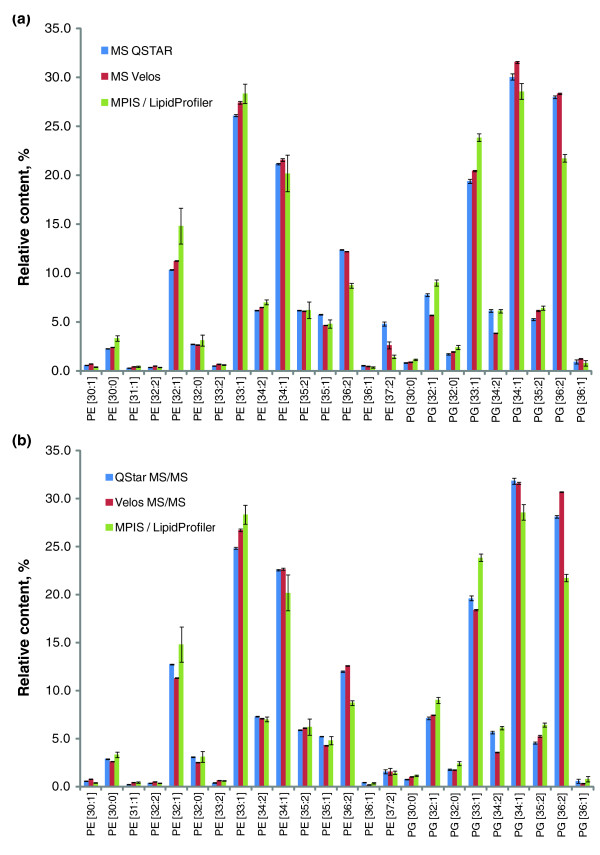
**LipidXplorer supports the interpretation of spectra acquired using different mass spectrometers**. **(a) **Comparison of the relative abundances of 24 major PE and PG lipid species identified in a total *E. coli *extract in MS and data-dependent MS/MS modes on the LTQ Orbitrap Velos (red bars) and QSTAR Pulsar *i *(blue bars) mass spectrometers, while spectra were interpreted by LipidXplorer. The same extract was analyzed by MPIS on the QSTAR Pulsar *i *and LipidProfiler software (green bars). Species abundances were normalized to the total abundance of the lipid class; error bars (standard deviation) were calculated on the basis of six experiments. Correlation coefficients and slopes of scatter plots for each pair-wise comparison are presented in Table 5.

We observed that the relative abundances of species quantified in MS and MS/MS spectra acquired on the Obitrap and QSTAR instruments by LipidXplorer were highly correlated and also corroborated the profile independently obtained by MPIS analysis and LipidProfiler software (Figure 10 and Table 5). We then correlated relative abundances of individual species determined by LipidXplorer in MS and MS/MS spectra acquired using different machines and different modes (for example, Orbitrap MS versus QSTAR MS/MS or Orbitrap MS/MS versus QSTAR MS) and compared them to profiles acquired on the same machine in different modes (Orbitrap MS versus Orbitrap MS/MS or QSTAR MS versus QSTAR MS/MS) (Additional file 14). In all independent comparisons (Figure 10; Additional file 14) we observed good correlation of relative quantities of individual lipid species. Importantly, the slopes of scatter plots were all close to a value of 1.0, indicating that LipidXplorer introduced no instrument-dependent or method-dependent systematic bias.

**Table 5:** Cross-platform correlation of relative abundances of *E. coli *lipids^a^

	Statistical	**Orbitrap versus QSTAR**^**c**^		**Orbitrap versus MPIS QSTAR**^**d**^		**QSTAR versus MPIS QSTAR**^**e**^	
Mode	Estimates^b^	PE	PG	PE	PG	PE	PG
MS	Correlation coefficient	0.99	0.99	0.97	0.94	0.95	0.94
	Slope	0.95	1.14	1.0	0.85	1.03	0.89
MS/MS	Correlation coefficient	0.99	0.99	0.97	0.94	0.95	0.94
	Slope	0.96	0.96	1.00	0.85	1.03	0.89

^a^Relative abundances of PE and PG species are presented in Figure 10. ^b^The values of correlation coefficients R^2 ^and slopes were calculated from corresponding scatter plots. ^c^Species were quantified by LipidXplorer using MS and MS/MS spectra acquired independently on the Orbitrap and QSTAR, respectively. MS/MS experiments were performed in data-dependent acquisition mode. ^d^Species were quantified by LipidXplorer using, respectively, MS and MS/MS spectra acquired at the Orbitrap machine. Relative abundances of individual species were correlated against corresponding relative abundances independently determined by MPIS on the QSTAR and LipidProfiler software. ^e^Species were quantified by LipidXplorer using, respectively, MS and MS/MS spectra acquired on the QSTAR machine in data-dependent acquisition mode. Relative abundances of individual species were correlated with those determined by MPIS and LipidProfiler.

We therefore concluded that LipidXplorer processed spectra acquired using different mass spectrometers and by different (MS and MS/MS) methods in a consistent and quantitative manner.

#### LipidXplorer exploits the diversity of lipid fragmentation pathways

Lipid identification relies upon specific 'signature' ions detectable in MS and/or MS/MS mode that, not necessarily unequivocally, distinguish the molecular species from molecules of other lipid classes or of the same class. The conceptual advance of MFQL is that many of these ions and/or their combinations can be simultaneously recognized in each MS/MS spectrum and bundled with several independent sc-constraints. Here we demonstrate that these assignments are accurate and coherent and could be employed in parallel to recognize individual species of multiple lipid classes in total lipid extracts.

A dataset of MS and MS/MS spectra was acquired in six technical replicates from a commercially available bovine heart total lipid extract on a LTQ Orbitrap XL mass spectrometer in negative ion mode. Using LipidXplorer software, a MasterScan database was compiled and probed with MFQL queries composed for 19 lipid classes, and 188 lipids of 15 classes were identified (Table 6). MFQL queries and the full list of identified species are provided in Additional files 15 and 16, respectively.

**Table 6:** Multifaceted identification of bovine brain lipid species by LipidXplorer

**Lipid class**^**a**^	Number of identified species	Number of signature ions	**FA**^**b**^	**FAO**^**b**^	**HG**^**b**^	**NL**^**b**^	**MS**^**c**^
PC	13	4	X X^d^			X	X
PC-*O*	17	4	X	X		X	X
LPC	4	3	X			X	X
Cer	3	2					X X
CL	10	1					X
LCL	2	2					X X
DAG	13	1					X
PE	22	3	X X				X
PE-*O*	35	3	X	X			X
LPE	4	2	X				X
PG	10	3	X X				X
PI	13	4	X X		X		X
PS	10	4	X X			X	X
SM	7	2				X	X
TAG	25	1					X

^a^MFQL queries identifying species of these lipid classes are presented in Additional file 15. ^b^FA, acyl anions of fatty acid moieties; FAO, product of the neutral loss of a fatty acid moiety from *sn-2 *position of the glycerol backbone; HG, fragment of the head group specific for all species of the same lipid class; NL, neutral loss of a fragment specific for all species of the same class; MS, precursor mass. ^c^Two X symbols indicate that precursor species of this class are detected in two molecular forms (for example, deprotonated ion and acetate adduct), or as doubly and singly charged ions. ^d^Two X symbols indicate that two signature ions of the same type are observed (for example, two acyl anions of both fatty acid moieties). Cer, ceramides; CL, cardiolipins; DAG, diacylglycerols; LCL, triacyl-lysocardiolipins; LPC, lysophosphatidylcholines; LPE; lyso-phosphatidylethanolamines; PC, phosphatidylcholines; PC-*O*, 1-alkyl-2-acylglycerophosphocholines; PE, phosphatidylethanolamines; PE-*O*, 1-alkyl-2-acylglycerophosphoethanolamines; PG, phosphatidylglycerols; PI, phosphatidylinositols; PS, phosphatidylserines; SM, sphingomyelins; TAG, triacylglycerols.

The interepretation of a shotgun dataset by LipidXplorer takes advantage of independent use of several signature ions for each lipid class. If detected at the high mass resolution, precursor ions of intact lipids are signature ions themselves. Some lipid classes, such as TAG, DAG and CL, have unique compositions of N, O and P atoms and can be unequivocally identified solely by their intact masses with no recourse to MS/MS [26].

**Figure 11:**
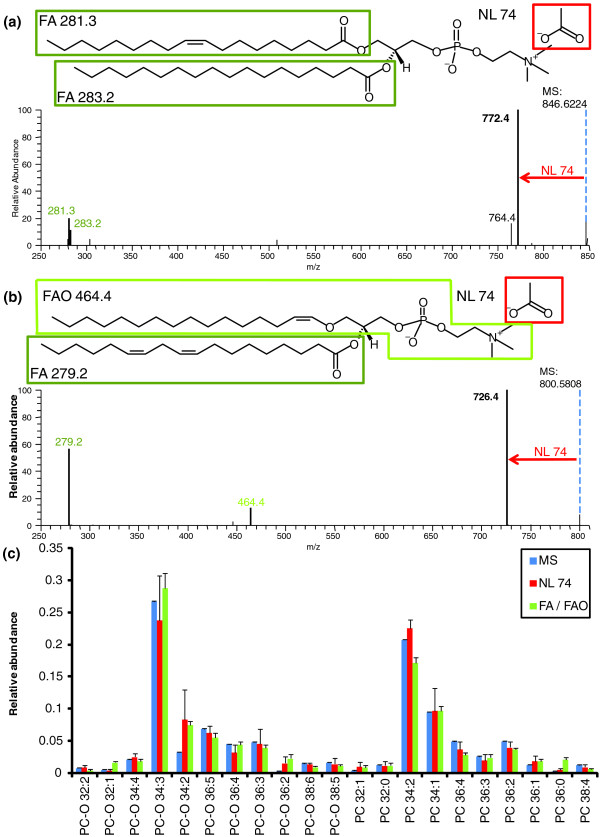
**Identification of PC and PC-*O *species by MFQL queries relying on complementary signature ions**. **(a) **MS/MS spectrum of the precursor ion of the acetate adduct of PC 36:1 (*m/z *846.6224), in which four signature ions are recognized: molecular ion (MS); fragment of neutral loss of acetate and methyl group (*Δm/z *= 74.0 (NL)); acyl anions of the two fatty acid moieties (FA 281.3 and FA 283.2; both boxed in the chemical structure at the top). **(b) **MS/MS spectrum of the acetate adduct of PC-*O *34:3 (*m/z *800.5808). Signature ions are the same as in (a), except *m/z *464.4 representing the fragment produced by neutral loss of the *sn-2 *fatty acid moiety. **(c) **Quantitative profiles of PC and PC-*O *species reported from abundances of different signature ions. MS, precursor ions in MS spectra; NL 74, neutral loss *Δm/z *74 in MS/MS spectra; FA/FAO, acyl anions of fatty acid moieties and (for PC-*O*) neutral loss of *sn-2 *fatty acid moiety. The relative abundance of species was normalized to the total abundance of species within each (PC or PC-*O*) class.

Otherwise, species identification should rely on signature ions in MS/MS spectra, such as acyl anions of fatty acid moieties, products of neutral losses of fatty acid moieties, head group fragments, and so on. As an example, we demonstrate here how using multiple signature ions helped in identifying molecular species of structurally related PC and PC-*O *lipids (Figure 5). The analysis was performed in negative ion mode in which both PC and PC-*O *were detected as molecular adducts with acetate anions (Figure 11). Species of both classes have the phosphorylcholine head group attached to the glycerol backbone at the *sn-3 *position and the fatty acid moiety at the *sn-2 *position (Figure 5). However, at the *sn-1 *position ester (PC) species have another fatty acid moiety, whereas ether (PC-*O*) species have a fatty alcohol moiety. To identify PC species, four signature ions could be considered (Table 6, Figure 11): intact precursor ion; fragment ion produced by neutral loss of 74 Da that is specific for the head group fragment; and two acyl anions of fatty acid moieties (Figure 11a). PC-*O *species can be identified (and distinguished from PC species) also by four signature ions (Table 6). Compared to PC, accurate masses of the intact precursor ion and of the fragment of 74 Da neutral loss should meet different sc-constraints. The third signature ion is the fragment of neutral loss of the fatty acid moiety and the fourth is the acyl anion of the fatty acid moiety itself (Figure 11b). At the same time, masses of the fatty acid and fatty alcohol moieties should complement the intact precursor mass. The high mass resolution of the Orbitrap mass analyzer allowed us to distinguish peaks of intact isobaric PC and PC-*O*, as well as of SM (also present in the total extract) and first isotopic peaks of PC. In MS/MS spectra we could clearly distinguish peaks of neutral loss products from co-selected PC, PC-*O *and SM precursors.

We note that signature ions could be recognized by MFQL queries even if fragments originating from accidentally co-fragmented precursors are also present. Users also have full flexibility to choose the signature ions and sc-constraints for species identification and alter MFQL queries accordingly, while the species profiles produced by alternative interepretations remain quantitatively consistent (Figure 11c).

Probing the MasterScan with correspondent MFQL queries effectively emulated several lipid class-specific and lipid species-specific precursor ion and neutral loss scans [10,16,36,37] (Figure 11). Signature ions might be associated with any structural feature of a lipid molecule and the power of the MFQL concept is that any of these can be recognized and used for the identification and quantification of individual species. Therefore, we argue that a combination of MFQL-assisted interpretation and the organization of shotgun lipidomics datasets in a MasterScan database enables cross-platform, accurate and comprehensive lipidomics analysis of complex biological samples.

## Conclusions

This study addresses the architecture, algorithms, validation and advanced features of LipidXplorer software, which supports the broadest scope of current shotgun lipidomics experiments, from targeted quantification of selected lipid species or classes to high-throughout lipidomics screens. LipidXplorer and its early prototype, LipidX, have been extensively tested in real-life applications and have already contributed interesting biological results [25,38-42]. Two key features distinguish LipidXplorer from other lipidomics software. First, the entire dataset comprising hundreds of MS and MS/MS spectra, including multiple technical and biological replicates, is organized into a single flat file database - the MasterScan. Second, for the first time, lipids are identified using user-defined queries formulated in the molecular fragmentation query language (MFQL). We demonstrate that MasterScan and MFQL make a powerful alliance enabling exhaustive interpretation of large shotgun datasets.

Shotgun lipidomics experiments could run on any tandem mass spectrometer with minimal sample preparation. We argue that, with flexible cross-platform software like LipidXplorer, a broad cell biology community can adopt lipidomics approaches for their specific needs, presumably at the same magnitude as proteomics methods are currently used. We note that LipidXplorer is just one possible implementation of a generic informatics concept that relies on MFQL-type interpretation of spectra. One much anticipated development is to extend the coverage of lipidomes of important model and medically relevant organisms by developing and validating queries covering all major lipid classes. An accessible public library of organism-specific queries should become an important resource for a broad lipidomics community. Better algorithms supporting all aspects of data processing could enhance the software potential in lipidomics screens. Importantly, LipidXplorer is an open-source software and its modular organization offers opportunity for further developments within a network of collaborating laboratories.

By eliminating major technical obstacles in identifying and quantifying any detectable lipid, LipidXplorer development revealed a few conceptual problems common to the entire lipidomics field. First, statistical estimates of species identification confidence should now be introduced also in lipidomics and each lipid composition report should be supported with a false discovery rate or similar statistical measure. It has become apparent (also from Table 4 and Figure 9) that false positive identifications commonly occur even when analyzing a relatively simple dataset. The next challenge would be to develop a statistical model that estimates identification confidence in a dataset- and instrument-specific way.

Another informatics challenge is unifying shotgun and liquid chromatography (LC)-MS or LC-MS/MS driven lipidomics on a common software platform. At the moment these approaches seem to be developing almost in parallel, although there have been efforts to enhance the performance of shotgun analysis by pre-fractionation of lipids by LC-MS [43]. We argue that, because of its flexible architecture and spectra interpretation routines, LipidXplorer has the potential to develop into an integrated platform supporting a palette of lipidomics applications in a consistent, statistically rigorous manner.

## Materials and methods

### Annotation of lipid species

Lipid classes are: PE, phosphatidylethanolamines; LPE; lyso-phosphatidylethanolamines; PE-*O*, 1-alkyl-2-acylglycerophosphoethanolamines; PS, phosphatidylserines; PC, phosphatidylcholines; PC-*O*, 1-alkyl-2-acylglycerophosphocholines; LPC, lysophosphatidylcholines; SM, sphingomyelins; PA, phosphatidic acids; PG, phosphatidylglycerols; PI, phosphatidylinositols; DAG, diacylglycerols; TAG, triacylglycerols; CL, cardiolipins; LCL, triacyl-lysocardiolipins; Cer, ceramides; Chol, cholesterol; CholEst, cholesterol esters.

Individual molecular species are annotated as follows: <lipid class > <no. of carbon atoms in the first fatty acid or fatty alcohol moiety >:<no. of double bonds in the first fatty acid or fatty alcohol moiety >/<no. of carbon atoms in the second fatty acid moiety >:<no. of double bonds in the second fatty acid moiety >. For example, PC 18:0/18:1 stands for a phosphatidylcholine comprising the moieties stearic (18:0) and oleic (18:1) fatty acids. If the exact composition of fatty acid or fatty alcohol moieties is not known, the species are annotated as: <lipid class > <no. of carbon atoms in both moieties >:<no. of double bonds in both moieties >. In this way, PC 36:1 stands for a PC species having 36 carbon atoms and one double bond in both fatty acid moieties.

### Mass spectrometry experiments

Mass spectrometry experiments were performed on a LTQ Orbitrap XL hybrid mass spectrometer (Thermo Fisher Scientific, Bremen, Germany) and, where specified, on a modified QSTAR Pulsar *i *quadrupole time-of-flight mass spectrometer (MDS Sciex, Concord, Ontario, Canada), both equipped with a robotic nanoflow ion source TriVersa (Advion BioSciences, Ithaca, NY, USA). If not specified otherwise, data-dependent acquisition was performed as described in [10]. A data-dependent acquisition cycle consisted of one MS spectrum followed by MS/MS spectra acquired from ten most abundant precursor ions, whose masses were subsequently excluded from further MS/MS experiments. MS/MS spectra were acquired on a LTQ Orbitrap using pulsed Q collision-induced dissociation (PQD) under the normalized collision energy of 21%. Fragment ions were detected at the linear ion trap (IT) or Orbitrap analyzers, as indicated separately for each experiment. The linear ion trap was operated at the low (unit) mass resolution *R*, while mass resolution of the Orbitrap was set for each experiment separately using the target resolution parameter specified as FWHM of the peak at *m/z *400. Where specified, LTQ Orbitrap MS/MS spectra were acquired by the method of higher energy collision-induced dissociation (HCD). Precursor ions were isolated by the linear ion trap at the unit resolution, fragmented in the HCD cell under the normalized collision energy of 45% and fragment ions detected by the Obitrap analyzer at a mass resolution of 7,500. MPIS scans were acquired on a quadrupole time-of-flight mass spectrometer QSTAR Pulsar *i *(AB Sciex, Toronto, Ontario, Canada) and interpreted by LipidProfiler software as described in [16]. Data-dependent MS/MS experiments on a QSTAR Pulsar *i *were performed as described in [10].

### Implementation of LipidXplorer software

LipidXplorer was programmed in Python 2.6. It imports spectra in *.mzXML [44] or peak lists in the *.dta/*.csv format. Free converters to *.mzXML are available at [45]. LipidXplorer automatically converts *.raw or *.wiff files into *.mzXML using, respectively, ReAdW or mzWiff programs.

LipidXplorer organizes mass spectra in a database-like format termed MasterScan (*.sc). The MasterScan is saved using Python's PICKLE function [46] for Python object serialization.

The MFQL interpreter is written using PLY (Python Lex-Yacc) [47], a lexer/parser generator based on Lex and Yacc. A collection of MFQL scripts is included in the distributed version of LipidXplorer and supports quantitative profiling of 19 major lipid classes. The routine for calculating sum compositions is an exhaustive search algorithm written in C and imported into Python.

The algorithm for calculating isotopic distributions was developed by Dr Magnus Palmblad (University of Reading, UK) and converted to Python by Dr Brian H Clowers using the NUMPY module [48].

LipidXplorer is available under general public license (GPL) at [49]. Full documentation on LipidXplorer, including the installation guidelines, a lipid identification tutorial and a library of MFQL scripts are provided at [50]. A sample dataset of shotgun mass spectra is also available for testing local installations of the software.

### LipidXplorer benchmarking: the dataset

*E. coli *total lipid extract was purchased from Avanti Polar Lipids (Alabaster, AL, USA) and analyzed on the LTQ Orbitrap XL instrument in negative ion mode. A solution of the total lipid concentration of 2.5 μg/ml in 7.5 mM ammonium acetate in choloroform/methanol/2-propanol (1/2/4, v/v/v) was infused into the mass spectrometer by TriVersa robotic ion source using a chip with the diameter of spraying nozzles of 4.1 μm. To produce the spectra dataset, the extract was analyzed in several independent experiments: experiment I, eight acquisitions under the unit mass resolution (*R*) settings using ion trap (IT) to acquire both MS and MS/MS spectra; experiment II, six acquisitions with *R *= 7,500 for MS spectra (Orbitrap) and unit resolution for MS/MS spectra (IT); experiment III, four acquisitions with *R *= 30,000 for MS spectra (Orbitrap) and unit resolution for MS/MS spectra (IT); experiment IV, four acquisitions with *R *= 100,000 for MS spectra (Orbitrap) and unit resolution for MS/MS spectra (IT); experiment V, seven acquisitions with *R *= 100,000 for MS spectra (Orbitrap) and *R *= 15,000 for MS/MS spectra (Orbitrap).

In the experiments I to IV, each acquisition produced approximately 33 MS and 330 MS/MS spectra; in the experiment V, 10 MS and 100 MS/MS spectra were acquired. To reduce undersampling, in the experiment V, acquisition of MS/MS spectra was navigated by the inclusion list compiled from 40 masses of plausible PE, PG and PA precursors A list of molecular lipid species was produced by manual interpretation of spectra acquired in the experiment V with requested mass tolerance of better than 3 ppm for precursors and 5 ppm for specific fragment ions. Only lipid species identified in at least four out of seven replicated analyses were included.

Spectra acquired in each of the experiments I to IV were further processed by LipidXplorer to produce corresponding MasterScan files. We used the dataset from the experiment I for comparative benchmarking of LipidXplorer against LipidQA and LipidSearch programs. Since LipidQA and LipidSearch do not align the spectra from replicated analyses, each acquisition was processed independently and then a non-redundant list of all identified lipid species was compiled.

### LipidXplorer benchmarking: the procedure

Eight acquisitions containing complete sets of MS and MS/MS spectra were independently submitted as *.raw files. The output was aligned by reported lipid species. Individual lipid species were considered as positively identified if they were recognized in four or more replicated analyses. In all tests the programs were prompted to identify species of PE, PI, PS, PG and PA classes. Mass tolerance was set at 0.3 Da in MS and MS/MS modes; fatty acid moieties were assumed to comprise 12 to 22 carbon atoms and 0 to 6 double bonds.

Settings specific for each tested program were as follows.

LipidXplorer: 'MS threshold' was set to 100 and 'MS/MS threshold' to 5 counts per peak area; 'Resolution gradient' was set to 1; other common spectra import settings were as in Additional file 13 (setting: 'FAS_LTQ').

LipidQA (spectra were imported as *.raw files): 'MS error' and the 'MS/MS error' were both set to 0.3 Da; 'Finnigan Filter', on; 'Quantification', off; 'Mode selection', Neg. Mode; 'If MS2 spectra were centroided', checked. Only species with a score above 0.5 were accepted. The current version of LipidQA is available at [51].

Lipid Search version 2.0 beta: 'SearchType' was set to 'MS2,MS3'; 'ExpType' to 'Infusion'; 'Precursor tol' to '0.3 Da'; 'Product peak tol' to 0.3 Da; 'Intensity threshold' to 0.01; 'Threshold type' to Relative; 'M-score Threshold' to 10.0. The current version of LipidSearch is available at [52].

LipidProfiler v.1.0.97: the software was used for creating a reference list of lipids in the *E. coli *extract and utilized a separate dataset acquired on a QSTAR Pulsar *i *mass spectrometer by the MPIS method. Intensity threshold was set to 0.2%; all lipid species reported as 'confirmed results' in at least four independent acquisitions.

### Validation of isotopic correction algorithm

We analyzed in two independent replicates a mixture of PA standards consisting of PA18:0/18:2, PA18:1/18:1, PA18:0/18:1 and PA18:0/18:0 (all from Avanti Polar Lipids) with the molar ratio of 1:9:1:1 on a LTQ Orbitrap Velos. Spectra were acquired under data-dependent acquisition control in negative mode using the linear ion trap analyzer under a target resolution of 800 for both MS and MS/MS. Precursors were fragmented using collision-induced dissociation. To process the dataset, mass tolerance was set to 300 ppm for MS and 500 ppm for MS/MS, spectra; occupation threshold was set to 0.5.

### Validation of the peak alignment algorithm

We used a dataset of 128 MS spectra of human blood plasma extracts acquired on a LTQ Orbitrap XL mass spectrometer. Spectra were imported into a MasterScan file assuming a mass resolution of 127,500 (FWHM, at *m/z *400), a mass accuracy of 4 ppm, and an occupation threshold of 0.5. Post-acquisition adjustment of peak masses was achieved using two reference masses of lipid standards spiked into the samples prior to extraction [25]. Lipids of 11 major classes (PC, PC-*O*, PE, PE-*O*,, LPC, LPE, SM, DAG, TAG, Chol and CholEst were identified by their accurate masses with no recourse to MS/MS.

### Validation of cross-platform quantification by LipidXplorer

Total lipid extract of *E. coli *was analyzed by multiple precursor ion scanning [16] and by data-dependent acquisition [10] on a QSTAR Pulsar *i *mass spectrometer. The same extract was analyzed by data-dependent HCD at the LTQ Orbitrap Velos mass spectrometer. Each analysis was performed in four replicates. Datasets of shotgun MS and MS/MS spectra were imported into MasterScan files built separately for each mass spectrometer and lipid species identified by MFQL queries (see Additional file 14 for the import settings and Additional file 11 for the queries). Lipid species were quantified in MS mode by using the intensities of their molecular ions. For MS/MS quantification, MFQL queries recognized and reported the sum of abundances of acyl anion fragments for each individual precursor. Relative quantities of individual lipids were calculated by normalizing to the total abundance of all species of the same lipid class. Parameters of linear correlation of lipid species profiles obtained by different methods (correlation coefficient R^2 ^and slope) were computed by Microsoft Excel (see Additional file 14).

### Analysis of bovine heart total lipid extract

Total lipid extract of bovine heart (Avanti Polar Lipids) was analyzed in six technical replicates on a LTQ-Orbitrap XL mass spectrometer using a target resolution of 100,000 for MS spectra (Orbitrap) and unit resolution for MS/MS (IT) in negative ion mode. Six replicates were acquired, each consisting of 31 MS and 310 MS/MS spectra.

### Publicly accessible depository of spectra

Mass spectra used for benchmarking and validating of LipidXplorer are available in original formats (*.raw for LTQ Orbitrap and *.wiff for QSTAR Pulsar *i*) at the LipidXplorer wiki page [50].

## Abbreviations

CL: cardiolipin; Chol: cholesterol; CholEst: cholesterol ester; DAG: diacylglycerol; FWHM: full width at half maximum; HCD: higher energy collision-induced dissociation; IT: ion trap; LC: liquid chromatography; MFQL: molecular fragmentation query language; MPIS: multiple precursorion scanning; MS: mass spectrometry; MS/MS: tandem mass spectrometry; PA: phosphatidic acid; PC: phosphatidylcholine; PE: phosphatidylethanolamine; PCF: Pearson correlation factor; PG: phosphatidylglycerol; PUFA: polyunsaturated fatty acid; sc: sum composition; SM: sphingomyelin; TAG: triacylglycerol.

## Authors' contributions

RH, DS and AS conceived the LipidXplorer concept, the study and wrote the manuscript. RH, DS and MS developed algorithms. RH wrote the software and validated it by computational experiments. DS, KS and JLS performed lipidomics experiments. SRB contributed samples for lipidomic analyses. All authors have read and approved the manuscript.

## Supplementary Material

Additional file 1 **Screenshots of the graphical user interface (GUI) of LipidXplorer**. Screenshots of four operational panels and explanations of their organization and available functionalities. Click here for file

Additional file 2 **Scan averaging algorithm**. A detailed mathematical description of the algorithm. Click here for file

Additional file 3 **Binning of peaks during scan averaging**. A figure showing a work scheme and explaining why the accuracy of average mass calculation improves with each binning cycle. Click here for file

Additional file 4 **Spectra alignment algorithm**. A detailed mathematical description of the algorithm. Click here for file

Additional file 5 **Common peak attributes considered by LipidXplorer**. Click here for file

Additional file 6 **Backus-Naur-Form (BNF) of the molecular fragmentation query language (MFQL)**. Click here for file

Additional file 7 **Comparison of 325 spectra independently averaged by Xcalibur and LipidXplorer**. A spreadsheet providing alignment details for each pair of spectra at different intensity thresholds. Click here for file

Additional file 8 **Validation of the isotopic correction algorithm**. A spreadsheet providing the abundances of peaks within partially overlapping isotopic clusters of PA lipids calculated with and without isotopic correction. Click here for file

Additional file 9 **Validation of the spectra alignment algorithm using a computationally generated spectra dataset**. A spreadsheet providing details of alignments of spectra processed using different numbers of binning cycles. Click here for file

Additional file 10 **Validation of the spectra alignment algorithm using MS spectra acquired from 128 total lipid extracts**. A spreadsheet providing a list of identified lipids and details of spectra alignment and correlation of peak intensities. Click here for file

Additional file 11 **MFQL scripts used for LipidXplorer benchmarking**. Click here for file

Additional file 12 **Benchmarking the LipidXplorer identification performance**. A spreadsheet providing lists of lipid species identified in a total *E. coli *extract by different software and their alignment with species from the reference list. Click here for file

Additional file 13 **Lipid species identified by LipidXplorer in spectra acquired at different mass resolution from a total *E. coli *extract**. A spreadsheet providing a list of species and intensities of their precursor and characteristic fragment ions in each individual spectrum. Click here for file

Additional file 14 **Validation of LipidXplorer cross-platform lipid identification by correlating relative abundances of *E. coli *lipid species determined in independent MS and MS/MS experiments on QSTAR Pulsar *i *and LTQ Orbitrap Velos mass spectrometers**. A spreadsheet providing LipidXplorer spectra processing settings, bar diagrams of relative abundances of individual species and statistical estimates of their correlation. Click here for file

Additional file 15 **MFQL scripts used for the lipid identification in a bovine heart total extract**. Click here for file

Additional file 16 **Lipid species identified by LipidXplorer in a total extract of bovine heart**. A spreadsheet providing a full list of lipid species and intensities of their precursor and characteristic fragment ions detected in independent MS and MS/MS experiments. Click here for file
