# Supplementary material for: A novel informatics concept for high-throughput shotgun lipidomics based on the molecular fragmentation query language
Source: Genome Biol. 2011 Jan 19;12(1):R8. doi: 10.1186/gb-2011-12-1-r8 (PMC3091306; doi:10.1186/gb-2011-12-1-r8)
Supplement: Additional file 1 — Screenshots of the graphical user interface (GUI) of LipidXplorer. Screenshots of four operational panels and explanations of their organization and available functionalities. [file gb-2011-12-1-r8-S1.PDF]

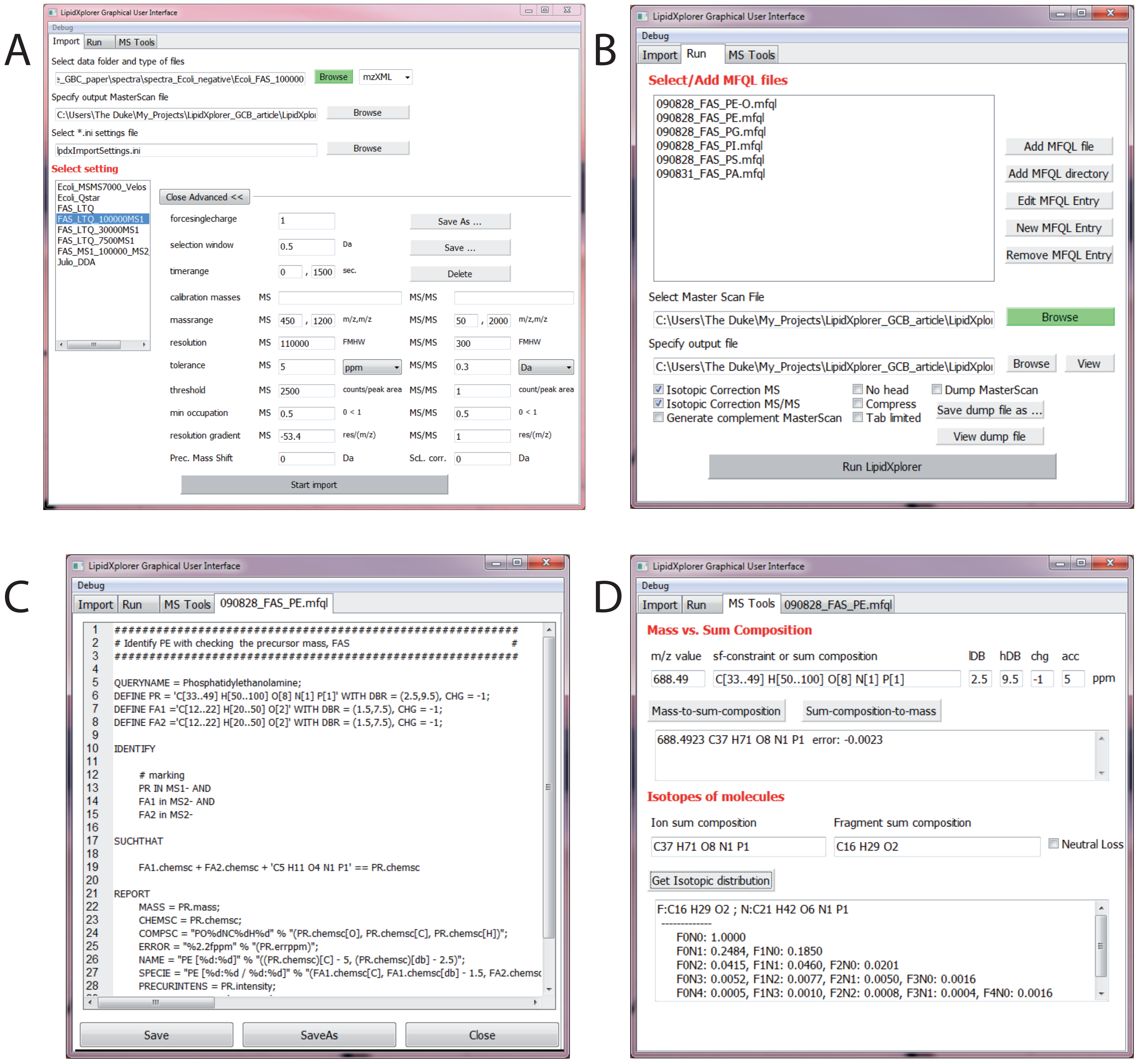

Figure S1 Screenshots of the LipidXplorer User Interface. A) A screenshot of the import panel. Sources for the import of the data are specified together with thier type (mzXML, csv/dta, centroided, profile). All import settings can be stored under a user specified name. B) A screenshot of the MFQL interpretation panel. One or more MFQL queries are selected for querying the imported MasterScan. Isotopic correction and other output options can be switched on and off. The result file and the content of the MasterScan can be viewed C) A screen-shot of the Editor panel. LipidXplorer has an integrated editor to edit and save MFQL queries. Several queries can be opened in parallel. D) A screenshot of the MS-Tools panel. This panel provides additional functions such as calculation of a sum composition using the compound mass, calculation the mass from a sum composition, calculation the isotopic cluster profile of the compound with known sum composition and/or its fragments.
