# Supplementary material for: A novel informatics concept for high-throughput shotgun lipidomics based on the molecular fragmentation query language
Source: Genome Biol. 2011 Jan 19;12(1):R8. doi: 10.1186/gb-2011-12-1-r8 (PMC3091306; doi:10.1186/gb-2011-12-1-r8)
Supplement: Additional file 2 — Scan averaging algorithm. A detailed mathematical description of the algorithm. [file gb-2011-12-1-r8-S2.PDF]

Detailed description of scan averaging algorithm

.1 Basic definitions

We introduce the notion of a spectrum used in this section:
A mass spectrum $S = \{p_0, ..., p_n\}$ is a set of peaks $p_i = (m_i, I_i, L_i)$ where m_i is the mass of the peak, I_i its intensity and L_i is the initially empty set of intensities for $i \in \{0, ..., n\}$. The abbreviation HWFM stands for Half Width Full Maximum.

.2 Spectra averaging of single scans

The inputs are:

- the single scan survey spectra given as peak lists S_i with $i = 1, .., n$,
- the resolution $R(m)$ at mass m. $R(m)$ is assumed to change linearly within the full mass range; its slope (mass resolution gradient) and intercept (resolution at the lowest mass of the full mass range) are instrument-dependent features pre-calculated by the user from some reference spectra
- the bin size of a given mass m $b(m) = \frac{m}{R(m)}$
- a threshold value T, representing a minimum intensity,
- an empty set S_{result}

First, all scans $S_1, ..., S_n$ are unioned to $\tilde{S}$, I.e. $\tilde{S} = \bigcup_{i=1}^{n} S_i$, suchthat $|\tilde{S}| = \sum_{i=1}^{n} |S_i|$. The peaks $p_1, ..., p_{|\tilde{S}|} \in \tilde{S}$, are sorted increasingly according the their mass. The algorithm begins with the smallest mass $p_i \in \tilde{S}$, where $i = 1$ initially :

1. repeat 3 times:

   i) collect all peaks, whose masses are not greater than $m_i + b(m_i)$ in a bin $B = \{p_i, p_{i+1}, ..., p_{i+k}\}$ for $k \in \mathbb{N}$

   ii) if there is at least one peak $p_i \in B$ whose intensity I_i is greater than T continue with iii), otherwise: go to iv)

   iii) calculāte the intensity weighted average $m_{avg} = \frac{\sum_{j=i}^{i+k} m_j \cdot I_j}{\sum_{j=i}^{i+k} I_j}$ of the masses, calculate the average intensity as $I_{scan} = \frac{\sum_{j=i}^{i+k} I_j}{k}$ and set $L_i = L_i \cup \{I_i, ..., I_{i+k}\}$. Store the result in the new spectrum $S_{new} = S_{new} \cup p_i$ where $p_i = (m_{avg}, I_{scan}, L_i)$

   iv) go to the successing peak of the greatest peak of the peaks in B, i.e. $i := i+k+1$ and continue the algorithm with Step i) till it reaches the end of $\tilde{S}$. If all peaks of $\tilde{S}$ are processed $\tilde{S} = S_{new}$

2. Calculate the resulting spectrum $S_{result} = \emptyset$ by calculating for every $p_i \in \tilde{S}$: $S_{result} = S_{result} \cup p_{result}$ where $p_{result} = (m_i, I_{scan})$ where $I_{scan} = \frac{\sum_{j \in L_i} j}{n}$ the average intensity over all scans.

3. Reduce spectrum by peaks which are below the threshold T. Since every scan $S_1, ..., S_n$ contains the information of the same sample, we can speak of a repeated acquisition. Thus, the signal-to-noise ratio increases when the scans are averaged. We account this behaviour by adjusting the user setted threshold value $T := \frac{T}{\sqrt{n}}$ due to the central limit theorem. We use this relation for reducing the spectrums noise, dependent on the number of scans. The noise reduction is so more adapted to the instrument. Let $S_{endresult} = \emptyset$ be the resulting spectrum:
for every peak $p_i = (m_i, I_i) \in S_{result}$ do:
if $I_i \geq \frac{T}{\sqrt{n}}$ then $S_{endresult} = S_{endresult} \cup p_i$
