# Supplementary material for: A novel informatics concept for high-throughput shotgun lipidomics based on the molecular fragmentation query language
Source: Genome Biol. 2011 Jan 19;12(1):R8. doi: 10.1186/gb-2011-12-1-r8 (PMC3091306; doi:10.1186/gb-2011-12-1-r8)
Supplement: Additional file 3 — Binning of peaks during scan averaging. A figure showing a work scheme and explaining why the accuracy of average mass calculation improves with each binning cycle. [file gb-2011-12-1-r8-S3.PDF]

Work scheme of the binning process in scan averaging

The peak distribution is assumed to be gaussian and leads to three times repeated averaging/alignment

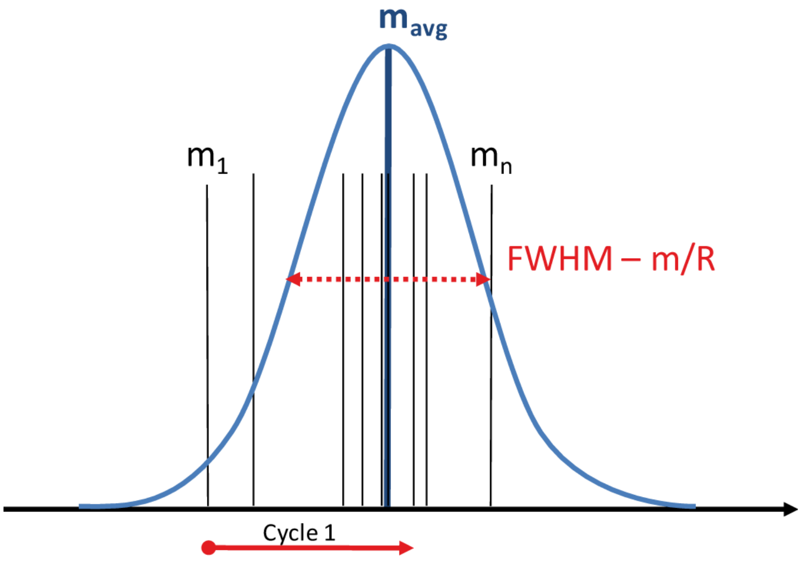

Figure : In a collapsed peak list the centroid masses ($m_1 - m_n$) of all scans (n) fall within a cluster. In the binning process we assume that all $m_1, m_2, \ldots, m_n$ are normal distributed around the true m_{avg}. This distribution is sampled by its FWHM which can be determined from $\frac{m}{R}$. In the first circle the smallest mass m_1 is applied to determine the start of the bin. Because of the determination of the higher bin border as $m_1 + \frac{m}{R}$ the first determined averaged mass of the first cycle (Cycle 1) bin must not reflect the complete distribution measured masses of that cluster.
