# Supplementary material for: A novel informatics concept for high-throughput shotgun lipidomics based on the molecular fragmentation query language
Source: Genome Biol. 2011 Jan 19;12(1):R8. doi: 10.1186/gb-2011-12-1-r8 (PMC3091306; doi:10.1186/gb-2011-12-1-r8)
Supplement: Additional file 4 — Spectra alignment algorithm. A detailed mathematical description of the algorithm. [file gb-2011-12-1-r8-S4.PDF]

Detailed description of spectra alignment algorithm

.1 Basic definitions

We introduce the notion of a spectrum:
A mass spectrum $S = \{p_0, ..., p_n\}$ is a set of peaks $p_i = (m_i, I_i, L_i)$ where m_i is the mass of the peak, I_i its intensity and L_i is the initially empty set of intensities for $i \in \{0, ..., n\}$. The abbreviation HWFM stands for Half Width Full Maximum.

.2 Alignment algorithm

The inputs are:

- all spectra S_i with $i = 1, .., n$ for all n samples,
- the resolution $R(m)$ at mass m. $R(m)$ is assumed to change linearly within the full mass range; its slope (mass resolution gradient) and intercept (resolution at the lowest mass of the full mass range) are instrument-dependent features pre-calculated by the user from some reference spectra
- the bin size of a given mass m $b(m) = \frac{m}{R(m)}$
- a minimum occupation threshold value C and
- an empty set S_{new}.

First, all scans $S_1, ..., S_n$ are summed to the spectrum $\tilde{S}$, I.e. $\tilde{S} = \bigcup_{i=1}^{n} S_i$ and $|\tilde{S}| = \sum_{i=1}^{n} |S_i|$. The peaks $p_1, ..., p_{|\tilde{S}|} \in \tilde{S}$ are sorted increasingly according the their mass. The algorithm begins with the smallest mass $p_i \in \tilde{S}$ where $i = 0$ initially:

1. repeat 3 times:
   i) collect all peaks, whose masses are not greater than $m_i + R$ in a bin $B = \{p_i, p_{i+1}, ..., p_{i+k}\}$
   ii) if there is at least one peak $p_i \in B$ whose intensity I_i is greater than T continue with iii), otherwise: go to iv)
   iii) calculate average $m_{avg} = \frac{\sum_{j=i}^{i+k} m_j \cdot I_j}{\sum_{j=i}^{i+k} I_j}$ of the masses, and $L_i = L_i \cup \{I_i, ..., I_{i+k}\}$. Store the result in the new spectrum $S_{new} = S_{new} \cup p_i$ where $p_i = (m_{avg}, I_{scan}, L_i)$
   iv) go to the successing peak of the greatest peak according to mass of B and continue the algorithm with Step i) till it reaches the end of $\tilde{S}$. If all peaks of $\tilde{S}$ are processed $\tilde{S} = S_{new}$

For $i \in |S_{new}|$, the resulting spectrum S_{new} contains now peaks $p_i = (m_i, L_i)$ with m_i is the average mass of all masses which where found in the samples and are within the bin size $b(m_i)$ and L_i is the set of the according intensities for every sample a mass was found.
