# Supplementary material for: A novel informatics concept for high-throughput shotgun lipidomics based on the molecular fragmentation query language
Source: Genome Biol. 2011 Jan 19;12(1):R8. doi: 10.1186/gb-2011-12-1-r8 (PMC3091306; doi:10.1186/gb-2011-12-1-r8)
Supplement: Additional file 5 — Common peak attributes considered by LipidXplorer. [file gb-2011-12-1-r8-S5.PDF]

0.1 List of peak attributes

Whenever we speak of neutral loss we address the neutral loss with the precursor ion.

- `mass`: the m/z value of the peak
- `chemsc`: the sum composition of the peak.
- `frsc`: the sum composition of the fragment. If the peak is a fragment, it is the same as `chemsc`, if it is a neutral loss, it returns the sum composition of the fragment.
- `nlsc`: the sum composition of the neutral loss. If the peak is a neutral loss, it is the same as `chemsc`, if it is a fragment, it returns the sum composition of the neutral loss.
- `frmass`: the mass of the fragment. If the peak is a fragment, it is the same as `mass`, if it is a neutral loss, it returns the mass of the fragment.
- `nlmass`: the mass of the neutral loss. If the peak is a neutral loss, it is the same as `mass`, if it is a fragment, it returns the mass of the neutral loss.
- `errppm`: is the difference between the exact mass and the measured mass in ppm
- `errda`: the error in Da
- `errres`: $= \frac{m}{\texttt{errda}}$ where m is the peaks mass
- `intensity`: is the list of all intensities through all given samples
