# Supplementary material for: A novel informatics concept for high-throughput shotgun lipidomics based on the molecular fragmentation query language
Source: Genome Biol. 2011 Jan 19;12(1):R8. doi: 10.1186/gb-2011-12-1-r8 (PMC3091306; doi:10.1186/gb-2011-12-1-r8)
Supplement: Additional file 6 — Backus-Naur-Form (BNF) of the molecular fragmentation query language (MFQL). [file gb-2011-12-1-r8-S6.PDF]

Backus-Naur-Form (BNF) of the Molecular Fragmentation Query Language (MFQL)

In the following we describe the syntax of MFQL with a BNF (Backus-Naur-Form) diagram. For readability we use regular expressions for the syntax of the tokens.

.1 MFQL tokens

id → `[A-z][0-9A-z]*`
sumComposition → `'([CHNOPDS][ia]?[0-999])+'`
sfConstrain → `'([CHNOPDS][ia]?\[[0-999]\]..\[[0-999]\])+'`
value → `[+-]?[1-9][0-9]*(\.[0-9]+)?`
stringWithPlaceholders → "*string*" see below

The token *stringWithPlaceholders* can be every string besides quotation marks and Python keywords. If the following strings occure in *string* they have the function of a placeholder:

- *%d* for a decimal value
- *%m.nf* for a floating point value, where m is the number of digits on the left side of the decimal point and n the number of digits on the right side of the decimal point.
- *%s* for a string value

.2 MFQL BNF diagram

⟨start⟩ ::⇒ ⟨variables⟩ , IDENTIFY ⟨identify⟩

⟨definitions⟩ ::⇒ ⟨definition⟩ ";" | ⟨definitions⟩ ";"
⟨definition⟩ ::⇒ *id* = ⟨content⟩
⟨content⟩ ::⇒ (*sumComposition* | *scConstraint* | *value*)

⟨identify⟩ ::⇒ ⟨identification⟩ | (SUCHTHAT ⟨suchthat⟩ | REPORT ⟨report⟩)
⟨identification⟩ ::⇒ ⟨scan⟩ | (AND | OR) ⟨identification⟩
⟨scan⟩ ::⇒ *id* IN MS(1 | 2)(+ | -)

⟨suchthat⟩ ::⇒ ⟨conditions⟩ REPORT ⟨report⟩
⟨conditions⟩ ::⇒ ⟨condition⟩ | (AND | OR) ⟨conditions⟩

⟨condition⟩ ::⇒ ⟨equation⟩ | ($<$ | $<=$ | $>$ | $>=$ | $==$) ⟨condition⟩
⟨equation⟩ ::⇒ ⟨term⟩ | (($+$ | $-$ | $*$ | $/$) ⟨equation⟩)
⟨term⟩ ::⇒ ⟨variable⟩ | ⟨function⟩ | *value* | *id*
⟨variable⟩ ::⇒ *id* | (*id*"["*id*"]") | (*id*"."*id*) | (*id*"."*id*"["*id*"]")

$\langle$function$\rangle$ ::$\Rightarrow$ *id*"("$\langle$attributes$\rangle$")"
$\langle$attributes$\rangle$ ::$\Rightarrow$ $\langle$term$\rangle$ | "," $\langle$attributes$\rangle$

$\langle$report$\rangle$ ::$\Rightarrow$ $\langle$mask$\rangle$ ";" | $\langle$report$\rangle$ ";"
$\langle$mask$\rangle$ ::$\Rightarrow$ *id* = $\langle$equation$\rangle$ | $\langle$term$\rangle$ | $\langle$string$\rangle$

$\langle$string$\rangle$ ::$\Rightarrow$ *stringWithPlaceholders* "%" "(" $\langle$attributes$\rangle$ ")"
