# Supplementary material for: A novel informatics concept for high-throughput shotgun lipidomics based on the molecular fragmentation query language
Source: Genome Biol. 2011 Jan 19;12(1):R8. doi: 10.1186/gb-2011-12-1-r8 (PMC3091306; doi:10.1186/gb-2011-12-1-r8)
Supplement: Additional file 11 — MFQL scripts used for LipidXplorer benchmarking. [file gb-2011-12-1-r8-S11.PDF]

```
########################################################
# Identify Ceramides with checking  the precursor mass   #
########################################################


QUERYNAME = ceramides;
DEFINE PR = 'C[32..44] H[30..100] N[1] O[3]' WITH DBR = (1.5,3.5), CHG = -1;
DEFINE PRA = 'C[34..42] H[30..100] N[1] O[5]' WITH DBR = (1.5,3.5), CHG = -1;


IDENTIFY
      # marking
      PRA IN MS1- OR
       PR IN MS1-


REPORT
      MASS = "%4.4f" % "(PRA.mass)";
      CHEMSC = PRA.chemsc;
       ERROR = "%2.2fppm" % "(PRA.errppm)";
      NAME = "Cer [%d:%d]" % "((PRA.chemsc)[C]-2, (PRA.chemsc)[db] - 1.5)";
      PRECURINTENS = PRA.intensity;;


################ end script #################



########################################################
# Identify CLwith checking  the precursor mass -2 charge #
########################################################


QUERYNAME = Cardiolipin;
DEFINE PR = 'C[65..87] H[90..180] O[17] P[2]' WITH DBR = (5,16), CHG = -2;


IDENTIFY Cardiolipin WHERE
      # marking
      PR IN MS1- WITH TOLERANCE = 5 ppm


SUCHTHAT
       isOdd(PR.chemsc[C])


REPORT
      MASS = "%4.4f" % "(PR.mass)";
      CHEMSC = PR.chemsc;
       ERROR = "%2.2fppm" % "(PR.errppm)";
      NAME = "CL [%d:%d]" % "((PR.chemsc)[C] - 9, (PR.chemsc)[db] - 5)";
       PRECURINTENS = PR.intensity;;


################ end script #################



########################################################
# Identify DAG with checking  the precursor mass (as acetate adduct)#
########################################################

QUERYNAME = Diacylglycerol;
```

```
DEFINE PR = 'C[33..49] H[35..110] O[7]' WITH DBR = (2.5,9.5), CHG = -1;


IDENTIFY
       # marking
       PR IN MS1-


SUCHTHAT
       isOdd(PR.chemsc[C])


REPORT
       MASS = "%4.4f" % "(PR.mass)";
       CHEMSC = PR.chemsc;
       ERROR = "%2.2fppm" % "(PR.errppm)";
       NAME = "DAG [%d:%d]" % "((PR.chemsc)[C] - 5, (PR.chemsc)[db] - 2.5)";
       PRECURINTENS = PR.intensity;;


############### end script #################



##########################################################
# Identify LPA with checking  the precursor mass AND FA  #
##########################################################


QUERYNAME = lysoPhosphatidicAcid;
DEFINE PR = 'C[17..25] H[30..70] O[7] P[1]' WITH DBR = (1.5,7.5), CHG = -1;
DEFINE FA1 ='C[14..22] H[20..50] O[2]' WITH DBR = (1.5,7.5), CHG = -1;


IDENTIFY lysoPhosphatidicAcid WHERE
       # marking
       PR IN MS1- AND
       FA1 IN MS2-


SUCHTHAT
       isEven(FA1.chemsc[C]) AND
       FA1.chemsc + 'C3 H7 O5 P1' == PR.chemsc


REPORT
       MASS = "%4.4f" % "(PR.mass)";
       CHEMSC = PR.chemsc;
       ERROR = "%2.2fppm" % "(PR.errppm)";
       NAME = "LPA [%d:%d]" % "((PR.chemsc)[C] - 3, (PR.chemsc)[db] - 1.5)";
       PRECURINTENS = PR.intensity;
       FAS = FA1.intensity;;


############### end script #################



##########################################################
# Identify LPC with checking  the precursor mass, NLS, FAS#
##########################################################
```

```
QUERYNAME = lysoPhosphatidylcholine;
DEFINE PR = 'C[24..32] H[30..80] O[9] N[1] P[1]' WITH DBR = (1.5,7.5), CHG = -1;
DEFINE headPC = 'C[3] H[6] O[2]' WITH CHG = 0;
DEFINE FA1 ='C[14..22] H[20..50] O[2]' WITH DBR = (1.5,7.5), CHG = -1;


IDENTIFY lysoPhosphatidylcholine WHERE
	# marking
	PR IN MS1- AND
	FA1 in MS2- AND
	headPC in MS2- WITH TOLERANCE = 0.5 Da


SUCHTHAT
	 isEven(PR.chemsc[C]) AND
	isEven(FA1.chemsc[C]) AND
	FA1.chemsc + headPC.nlsc + 'C7 H16 P1 O5 N1' == PR.chemsc


REPORT
	MASS = "%4.4f" % "(PR.mass)";
	CHEMSC = PR.chemsc;
	 ERROR = "%2.2fppm" % "(PR.errppm)";
	NAME = "LPC [%d:%d]" % "((PR.chemsc)[C] - 10, (PR.chemsc)[db] - 1.5)";
	PRECURINTENS = PR.intensity;
	NLSPIS = headPC.intensity;
	FAS = FA1.intensity;;

################ end script ##################



##########################################################
# Identify LPE with checking  the precursor mass AND FA  #
##########################################################


QUERYNAME = lysoPhosphatidylethanolamine;
DEFINE PR = 'C[19..27] H[30..70] O[7] N[1] P[1]' WITH DBR = (1.5,7.5), CHG = -1;
DEFINE FA1 ='C[14..22] H[20..50] O[2]' WITH DBR = (1.5,7.5), CHG = -1;


IDENTIFY lysoPhosphatidylethanolamine WHERE
	# marking
	PR IN MS1- AND
	FA1 in MS2-


SUCHTHAT
	 isOdd(PR.chemsc[C]) AND
	isEven(FA1.chemsc[C]) AND
	FA1.chemsc + 'C5 H12 O5 N1 P1' == PR.chemsc


REPORT
	MASS = "%4.4f" % "(PR.mass)";
	CHEMSC = PR.chemsc;
	 ERROR = "%2.2fppm" % "(PR.errppm)";
	NAME = "LPE [%d:%d]" % "((PR.chemsc)[C] - 5, (PR.chemsc)[db] - 1.5)";
	PRECURINTENS = PR.intensity;
	FAS = FA1.intensity;;
```

```
################ end script #################



##########################################################
# Identify LPG with checking  the precursor mass AND FA  #
##########################################################


QUERYNAME = lysoPhosphatidylglycerol;
DEFINE PR = 'C[20..28] H[30..70] O[9] P[1]' WITH DBR = (1.5,7.5), CHG = -1;
DEFINE FA1 ='C[14..22] H[20..50] O[2]' WITH DBR = (1.5,7.5), CHG = -1;


IDENTIFY lysoPhosphatidylglycerol WHERE
       # marking
       PR IN MS1- AND
       FA1 IN MS2-


SUCHTHAT
       isEven(FA1.chemsc[C]) AND
       FA1.chemsc + 'C6 H13 O7 P1' == PR.chemsc


REPORT
       MASS = "%4.4f" % "(PR.mass)";
       CHEMSC = PR.chemsc;
       ERROR = "%2.2fppm" % "(PR.errppm)";
       NAME = "LPG [%d:%d]" % "((PR.chemsc)[C] - 6, (PR.chemsc)[db] - 1.5)";
       PRECURINTENS = PR.intensity;
       FAS = FA1.intensity;;

################ end script #################



##########################################################
# Identify PI with checking  the precursor mass          #
##########################################################


QUERYNAME = LysoPhosphatidylinositol;
DEFINE PR = 'C[37..53] H[30..140] O[12] P[1]' WITH DBR = (2.5,9.5), CHG = -1;
DEFINE headPI = 'C[6] H[10] O[8] P[1]' WITH DBR = (1.5,4.5), CHG = -1;
DEFINE FA1 ='C[14..22] H[20..50] O[2]' WITH DBR = (1.5,7.5), CHG = -1;


IDENTIFY LysoPhosphatidylinositol WHERE
       # marking
       PR IN MS1- AND
       FA1 in MS2- AND
       headPI in MS2-


SUCHTHAT
       isOdd(PR.chemsc[C]) AND
       isEven(FA1.chemsc[C]) AND
       FA1.chemsc +  headPI.chemsc + 'C3 H7 O2' == PR.chemsc
```

```
REPORT
	MASS = "%4.4f" % "(PR.mass)";
	CHEMSC = PR.chemsc;
	ERROR = "%2.2fppm" % "(PR.errppm)";
	NAME = "LPI [%d:%d]" % "((PR.chemsc)[C] - 9, (PR.chemsc)[db] - 2.5)";
	PRECURINTENS = PR.intensity;
	NLSPIS = headPI.intensity;
	FAS = FA1.intensity;;


################ end script ##################



##########################################################
# Identify lysoCLwith checking  the precursor mass -2 charge#
##########################################################


QUERYNAME = lysoCardiolipin;
DEFINE PR = 'C[51..69] H[90..180] O[16] P[2]' WITH DBR = (4,16), CHG = -2;
DEFINE PRA = 'C[51..69] H[90..180] O[16] P[2]' WITH DBR = (3.5,15.5), CHG = -1;


IDENTIFY lysoCardiolipin WHERE
	# marking
	PR IN MS1- WITH TOLERANCE = 5 ppm OR
	PRA IN MS1- WITH TOLERANCE = 5 ppm


REPORT
	MASS = "%4.4f" % "(PR.mass)";
	CHEMSC = PR.chemsc;
	ERROR = "%2.2fppm" % "(PR.errppm)";
	NAME = "LysCL [%d:%d]" % "((PR.chemsc)[C] - 9, (PR.chemsc)[db] - 4)";
	PRECURINTENS = PR.intensity;;


################ end script ##################



##########################################################
# Identify PA with checking  the precursor mass and FAS  #
##########################################################


QUERYNAME = PhosphatidicAcid;
DEFINE PR = 'C[31..47] H[30..120] O[8] P[1]' WITH DBR = (2.5,8.5), CHG = -1;
DEFINE FA1 ='C[14..22] H[20..50] O[2]' WITH DBR = (1.5,7.5), CHG = -1;
DEFINE FA2 ='C[14..22] H[20..50] O[2]' WITH DBR = (1.5,7.5), CHG = -1;


IDENTIFY PhosphatidicAcid WHERE
	# marking
	PR IN MS1- AND
	FA1 in MS2- AND
	FA2 in MS2-

SUCHTHAT
	isOdd(PR.chemsc[C]) AND
	isEven(FA1.chemsc[C]) AND
	isEven(FA2.chemsc[C]) AND
```

```
	FA1.chemsc + FA2.chemsc + 'C3 H6 P1 O4' == PR.chemsc


REPORT
	MASS = "%4.4f" % "(PR.mass)";
	CHEMSC = PR.chemsc;
	 ERROR = "%2.2fppm" % "(PR.errppm)";
	NAME = "PA [%d:%d]" % "((PR.chemsc)[C] - 3, (PR.chemsc)[db] - 2.5)";
	SPECIE = "PA [%d:%d / %d:%d]" % "(FA1.chemsc[C], FA1.chemsc[db] - 1.5,
FA2.chemsc[C], FA2.chemsc[db] - 1.5)";
	PRECURINTENS = PR.intensity;
	FAS = sumIntensity(FA1, FA2);;


################ end script ##################



##########################################################
# Identify PC with checking  the precursor mass, NLS, FAS#
##########################################################


QUERYNAME = Phosphatidylcholine;
DEFINE PR = 'C[38..54] H[30..130] O[10] N[1] P[1]' WITH DBR = (2.5,9.5), CHG = -1;
DEFINE headPC = 'C[3] H[6] O[2]' WITH CHG = 0;
DEFINE FA1 ='C[14..22] H[20..50] O[2]' WITH DBR = (1.5,7.5), CHG = -1;
DEFINE FA2 ='C[14..22] H[20..50] O[2]' WITH DBR = (1.5,7.5), CHG = -1;


IDENTIFY Phosphatidylcholine WHERE
	# marking
	PR IN MS1- AND
	FA1 in MS2- AND
	FA2 in MS2- AND
	headPC in MS2- WITH TOLERANCE = 0.5 Da


SUCHTHAT
	isEven(PR.chemsc[C]) AND
	isEven(FA1.chemsc[C]) AND
	isEven(FA2.chemsc[C]) AND
	FA1.chemsc + FA2.chemsc + headPC.nlsc + 'C7 H15 P1 O4 N1' == PR.chemsc


REPORT
	MASS = "%4.4f" % "(PR.mass)";
	CHEMSC = PR.chemsc;
	 ERROR = "%2.2fppm" % "(PR.errppm)";
	NAME = "PC [%d:%d]" % "((PR.chemsc)[C] - 10, (PR.chemsc)[db] - 2.5)";
	SPECIE = "PC [%d:%d / %d:%d]" % "(FA1.chemsc[C], FA1.chemsc[db] - 1.5,
FA2.chemsc[C], FA2.chemsc[db] - 1.5)";
	PRECURINTENS = PR.intensity;
	NLSPIS = headPC.intensity;
	FAS = sumIntensity(FA1, FA2);;


################ end script ##################



##########################################################
# Identify PC-O with checking  the precursor mass, NLS, FAS#
```

```
##########################################################


QUERYNAME = Phosphatidylcholineether;
DEFINE PR = 'C[38..54] H[30..120] O[9] N[1] P[1]' WITH DBR = (1.5,8.5), CHG = -1;
DEFINE headPC = 'C[3] H[6] O[2]' WITH CHG = 0;
DEFINE FA1 ='C[14..22] H[20..50] O[2]' WITH DBR = (1.5,7.5), CHG = -1;
DEFINE FAO ='C[21..29] H[20..80] O[6] N[1] P[1]' WITH DBR = (0.5,6.5), CHG = -1;


IDENTIFY Phosphatidylcholineether WHERE
	# marking
	PR IN MS1- AND
	FA1 in MS2- AND
	FAO in MS2- AND
	headPC in MS2- WITH TOLERANCE = 0.5 Da


SUCHTHAT
	isEven(PR.chemsc[C]) AND
	isEven(FA1.chemsc[C]) AND
	isOdd(FAO.chemsc[C]) AND
	FA1.chemsc + FAO.chemsc + headPC.nlsc == PR.chemsc + 'O1 H1'


REPORT
	MASS = "%4.4f" % "(PR.mass)";
	CHEMSC = PR.chemsc;
	 ERROR = "%2.2fppm" % "(PR.errppm)";
	NAME = "PC-O [%d:%d]" % "((PR.chemsc)[C] - 10, (PR.chemsc)[db] - 1.5)";
	SPECIE = "PC-O [%d:%d / %d:%d]" % "(FAO.chemsc[C] - 7, FAO.chemsc[db] - 0.5,
FA1.chemsc[C], FA1.chemsc[db] - 1.5)";
	PRECURINTENS = PR.intensity;
	NLSPIS = headPC.intensity;
	FAS = sumIntensity(FA1, FAO);;


################ end script #################



##########################################################
# Identify PE with checking  the precursor mass, FAS     #
##########################################################


QUERYNAME = Phosphatidylethanolamine;
DEFINE PR = 'C[33..49] H[50..100] O[8] N[1] P[1]' WITH DBR = (2.5,9.5), CHG = -1;
DEFINE FA1 ='C[14..22] H[20..50] O[2]' WITH DBR = (1.5,7.5), CHG = -1;
DEFINE FA2 ='C[14..22] H[20..50] O[2]' WITH DBR = (1.5,7.5), CHG = -1;


IDENTIFY Phosphatidylethanolamine WHERE
	# marking
	PR IN MS1- AND
	FA1 in MS2- AND
	FA2 in MS2-


SUCHTHAT
	isOdd(PR.chemsc[C]) AND
	isEven(FA1.chemsc[C]) AND
	isEven(FA2.chemsc[C]) AND
```

```
	FA1.chemsc + FA2.chemsc + 'C5 H11 O4 N1 P1' == PR.chemsc


REPORT
	MASS = "%4.4f" % "(PR.mass)";
	CHEMSC = PR.chemsc;
	 ERROR = "%2.2fppm" % "(PR.errppm)";
	NAME = "PE [%d:%d]" % "((PR.chemsc)[C] - 5, (PR.chemsc)[db] - 2.5)";
	SPECIE = "PE [%d:%d / %d:%d]" % "(FA1.chemsc[C], FA1.chemsc[db] - 1.5,
FA2.chemsc[C], FA2.chemsc[db] - 1.5)";
	PRECURINTENS = PR.intensity;
	FAS = sumIntensity(FA1, FA2);;


################ end script ##################



##########################################################
# Identify PE-O with checking  the precursor mass, FAS and FA-O determination #
##########################################################


QUERYNAME = Phosphatidylethanolamineether;
DEFINE PR = 'C[33..49] H[50..100] O[7] N[1] P[1]' WITH DBR = (1.5,8.5), CHG = -1;
DEFINE FA1 ='C[14..22] H[20..50] O[2]' WITH DBR = (1.5,7.5), CHG = -1;
DEFINE FAO ='C[19..27] H[20..80] O[6] N[1] P[1]' WITH DBR = (0.5,6.5), CHG = -1;


IDENTIFY Phosphatidylethanolamineether WHERE
	# marking
	PR IN MS1- WITH TOLERANCE = 2.5 ppm AND
	FA1 in MS2- WITH TOLERANCE = 0.3 Da AND
	FAO in MS2- WITH TOLERANCE = 0.3 Da


SUCHTHAT
	isOdd(PR.chemsc[C]) AND
	isEven(FA1.chemsc[C]) AND
	isOdd(FAO.chemsc[C]) AND
	FA1.chemsc + FAO.chemsc == PR.chemsc + 'O1 H1'


REPORT
	MASS = "%4.4f" % "(PR.mass)";
	CHEMSC = PR.chemsc;
	 ERROR = "%2.2fppm" % "(PR.errppm)";
	NAME = "PE-O [%d:%d]" % "((PR.chemsc)[C] - 5, (PR.chemsc)[db] - 1.5)";
	SPECIE = "PE-O [%d:%d / %d:%d]" % "(FAO.chemsc[C] - 5, FAO.chemsc[db] - 0.5,
FA1.chemsc[C], FA1.chemsc[db] - 1.5)";
	PRECURINTENS = PR.intensity;
	FAS = FA1.intensity + FAO.intensity;;


################ end script ##################



##########################################################
# Identify PG with checking  the precursor mass and FAS  #
##########################################################
```

```
QUERYNAME = Phosphatidylglycerol;
DEFINE PR = 'C[34..50] H[30..120] O[10] P[1]' WITH DBR = (2.5,8.5), CHG = -1;
DEFINE FA1 ='C[14..22] H[20..50] O[2]' WITH DBR = (1.5,7.5), CHG = -1;
DEFINE FA2 ='C[14..22] H[20..50] O[2]' WITH DBR = (1.5,7.5), CHG = -1;


IDENTIFY Phosphatidylglycerol WHERE
       # marking
       PR IN MS1-  AND
       FA1 in MS2- AND
       FA2 in MS2-


SUCHTHAT
        isEven(PR.chemsc[C]) AND
       isEven(FA1.chemsc[C]) AND
       isEven(FA2.chemsc[C]) AND
       FA1.chemsc + FA2.chemsc + 'C6 H12 P1 O6' == PR.chemsc


REPORT
       MASS = "%4.4f" % "(PR.mass)";
       CHEMSC = PR.chemsc;
        ERROR = "%2.2fppm" % "(PR.errppm)";
       NAME = "PG [%d:%d]" % "((PR.chemsc)[C] - 6, (PR.chemsc)[db] - 2.5)";
       SPECIE = "PG [%d:%d / %d:%d]" % "(FA1.chemsc[C], FA1.chemsc[db] - 1.5,
FA2.chemsc[C], FA2.chemsc[db] - 1.5)";
       PRECURINTENS = PR.intensity;
       FAS = sumIntensity(FA1, FA2);;


################ end script ##################



##########################################################
# Identify PI with checking  the precursor mass, FAs, Headfrag#
##########################################################


QUERYNAME = Phosphatidylinositol;
DEFINE PR = 'C[37..53] H[30..140] O[13] P[1]' WITH DBR = (3.5,10.5), CHG = -1;
DEFINE headPI = 'C[6] H[10] O[8] P[1]' WITH DBR = (1.5,4.5), CHG = -1;
DEFINE FA1 ='C[14..22] H[20..50] O[2]' WITH DBR = (1.5,7.5), CHG = -1;
DEFINE FA2 ='C[14..22] H[20..50] O[2]' WITH DBR = (1.5,7.5), CHG = -1;


IDENTIFY Phosphatidylinositol WHERE
       # marking
       PR IN MS1- AND
       FA1 in MS2- AND
       FA2 in MS2- AND
       headPI in MS2-


SUCHTHAT
        isOdd(PR.chemsc[C]) AND
       isEven(FA1.chemsc[C]) AND
       isEven(FA2.chemsc[C]) AND
       FA1.chemsc + FA2.chemsc + headPI.chemsc + 'C3 H6 O1' == PR.chemsc
```

```
REPORT
       MASS = "%4.4f" % "(PR.mass)";
       CHEMSC = PR.chemsc;
        ERROR = "%2.2fppm" % "(PR.errppm)";
       NAME = "PI [%d:%d]" % "((PR.chemsc)[C] - 9, (PR.chemsc)[db] - 3.5)";
       SPECIE = "PI [%d:%d / %d:%d]" % "(FA1.chemsc[C], FA1.chemsc[db] - 1.5,
FA2.chemsc[C], FA2.chemsc[db] - 1.5)";
       PRECURINTENS = PR.intensity;
       NLSPIS = headPI.intensity;
       FAS = FA1.intensity + FA2.intensity;;


################ end script ##################



##########################################################
# Identify PS with checking  the precursor mass, NLS and FAS#
##########################################################


QUERYNAME = Phosphatidylserine;
DEFINE PR = 'C[34..50] H[30..120] O[10] N[1] P[1]' WITH DBR = (3.5,10.5), CHG = -1;
DEFINE headPS = 'C[3] H[5] O[2] N[1]' WITH DBR = (-0.5,6.5), CHG = 0;
DEFINE FA1 ='C[14..22] H[20..50] O[2]' WITH DBR = (1.5,7.5), CHG = -1;
DEFINE FA2 ='C[14..22] H[20..50] O[2]' WITH DBR = (1.5,7.5), CHG = -1;


IDENTIFY Phosphatidylserine WHERE
       # marking
       PR IN MS1-  AND
       FA1 in MS2- AND
       FA2 in MS2- AND
       headPS in MS2- WITH TOLERANCE = 0.5 Da


SUCHTHAT
        isEven(PR.chemsc[C]) AND
       FA1.chemsc + FA2.chemsc + headPS.nlsc + 'C3 H6 P1 O4' == PR.chemsc


REPORT
       MASS = "%4.4f" % "(PR.mass)";
       CHEMSC = PR.chemsc;
        ERROR = "%2.2fppm" % "(PR.errppm)";
       NAME = "PS [%d:%d]" % "((PR.chemsc)[C] - 6, (PR.chemsc)[db] - 3.5)";
       SPECIE = "PS [%d:%d / %d:%d]" % "(FA1.chemsc[C], FA1.chemsc[db] - 1.5,
FA2.chemsc[C], FA2.chemsc[db] - 1.5)";
       PRECURINTENS = PR.intensity;
       NLSPIS = headPS.intensity;
       FAS = sumIntensity(FA1, FA2);;


################ end script ##################



##########################################################
# Identify SM with checking  the precursor mass, NLS     #
##########################################################
```

```
QUERYNAME = Sphingomyelin;
DEFINE PR = 'C[39..49] H[30..130] O[8] N[2] P[1]' WITH DBR = (1.5,3.5), CHG = -1;
DEFINE headSMPC = 'C[3] H[6] O[2]' WITH CHG = 0;


IDENTIFY Sphingomyelin WHERE
       # marking
       PR IN MS1- AND
       headSMPC in MS2- WITH TOLERANCE = 0.5 Da


SUCHTHAT
        isOdd(PR.chemsc[C])


REPORT
       MASS = "%4.4f" % "(PR.mass)";
       CHEMSC = PR.chemsc;
        ERROR = "%2.2fppm" % "(PR.errppm)";
       NAME = "SM [%d:%d]" % "((PR.chemsc)[C] - 7, (PR.chemsc)[db] - 1.5)";
       PRECURINTENS = PR.intensity;
       NLSPIS = headSMPC.intensity;;


################ end script ##################



##########################################################
# Identify TAG with checking  the precursor mass (as acetate adduct)#
##########################################################


QUERYNAME = Triacylglycerol;
DEFINE PR = 'C[47..65] H[50..160] O[8]' WITH DBR = (3.5,10.5), CHG = -1;


IDENTIFY Triacylglycerol WHERE
       # marking
       PR IN MS1- WITH TOLERANCE = 2.5 ppm


SUCHTHAT
        isOdd(PR.chemsc[C])


REPORT
       MASS = "%4.4f" % "(PR.mass)";
       CHEMSC = PR.chemsc;
        ERROR = "%2.2fppm" % "(PR.errppm)";
       NAME = "TAG [%d:%d]" % "((PR.chemsc)[C] - 5, (PR.chemsc)[db] - 3.5)";
       PRECURINTENS = PR.intensity;;


################ end script ##################
```
