# Supplementary material for: A novel informatics concept for high-throughput shotgun lipidomics based on the molecular fragmentation query language
Source: Genome Biol. 2011 Jan 19;12(1):R8. doi: 10.1186/gb-2011-12-1-r8 (PMC3091306; doi:10.1186/gb-2011-12-1-r8)
Supplement: Additional file 15 — MFQL scripts used for the lipid identification in a bovine heart total extract. [file gb-2011-12-1-r8-S15.PDF]

```
##########################################################
# Identify PE with checking  the precursor mass, FAS     #
##########################################################


QUERYNAME = Phosphatidylethanolamine;
DEFINE PR = 'C[33..49] H[50..100] O[8] N[1] P[1]' WITH DBR = (2.5,9.5), CHG = -1;
DEFINE FA1 ='C[12..22] H[20..50] O[2]' WITH DBR = (1.5,7.5), CHG = -1;
DEFINE FA2 ='C[12..22] H[20..50] O[2]' WITH DBR = (1.5,7.5), CHG = -1;


IDENTIFY
	# marking
	PR IN MS1- AND
	FA1 in MS2- AND
	FA2 in MS2-


SUCHTHAT
	FA1.chemsc + FA2.chemsc + 'C5 H11 O4 N1 P1' == PR.chemsc


REPORT
	MASS = "%4.4f" % "(PR.mass)";
	CHEMSC = PR.chemsc;
	COMPSC = "PO%dNC%dH%d" % "(PR.chemsc[O], PR.chemsc[C], PR.chemsc[H])";
	 ERROR = "%2.2fppm" % "(PR.errppm)";
	NAME = "PE [%d:%d]" % "((PR.chemsc)[C] - 5, (PR.chemsc)[db] - 2.5)";
	SPECIE = "PE [%d:%d / %d:%d]" % "(FA1.chemsc[C], FA1.chemsc[db] - 1.5,
FA2.chemsc[C], FA2.chemsc[db] - 1.5)";
	PRECURINTENS = PR.intensity;
	FAS = sumIntensity(FA1, FA2);;


################ end script #################




################################################################################
# Identify PE-O with checking  the precursor mass, FAS and FA-O determination #
################################################################################


QUERYNAME = Phosphatidylethanolamineether;
DEFINE PR = 'C[33..49] H[50..100] O[7] N[1] P[1]' WITH DBR = (1.5,8.5), CHG = -1;
DEFINE FA1 ='C[12..22] H[20..50] O[2]' WITH DBR = (1.5,7.5), CHG = -1;
DEFINE FAO ='C[17..27] H[20..80] O[6] N[1] P[1]' WITH DBR = (0.5,6.5), CHG = -1;


IDENTIFY Phosphatidylethanolamineether WHERE
	# marking
	PR IN MS1-  AND
	FA1 in MS2-  AND
	FAO in MS2-


SUCHTHAT
	FA1.chemsc + FAO.chemsc == PR.chemsc + 'O1 H1'


REPORT
	MASS = "%4.4f" % "(PR.mass)";
```

```
	CHEMSC = PR.chemsc;
	COMPSC = "PO%dNC%dH%d" % "(PR.chemsc[O], PR.chemsc[C], PR.chemsc[H])";
	 ERROR = "%2.2fppm" % "(PR.errppm)";
	NAME = "PE-O [%d:%d]" % "((PR.chemsc)[C] - 5, (PR.chemsc)[db] - 1.5)";
	SPECIE = "PE-O [%d:%d / %d:%d]" % "(FA0.chemsc[C] - 5, FA0.chemsc[db] - 0.5,
FA1.chemsc[C], FA1.chemsc[db] - 1.5)";
	PRECURINTENS = PR.intensity;
	FAS = FA1.intensity + FA0.intensity;;


################ end script ##################



##########################################################
# Identify PG with checking  the precursor mass and FAS         #
##########################################################


QUERYNAME = Phosphatidylglycerol;
DEFINE PR = 'C[34..50] H[30..120] O[10] P[1]' WITH DBR = (2.5,9.5), CHG = -1;
DEFINE FA1 ='C[12..22] H[20..50] O[2]' WITH DBR = (1.5,7.5), CHG = -1;
DEFINE FA2 ='C[12..22] H[20..50] O[2]' WITH DBR = (1.5,7.5), CHG = -1;


IDENTIFY Phosphatidylglycerol WHERE
	# marking
	PR IN MS1- AND
	FA1 in MS2-  AND
	FA2 in MS2-


SUCHTHAT
	FA1.chemsc + FA2.chemsc + 'C6 H12 P1 O6' == PR.chemsc


REPORT
	MASS = "%4.4f" % "(PR.mass)";
	CHEMSC = PR.chemsc;
	COMPSC = "PO%dC%dH%d" % "(PR.chemsc[O], PR.chemsc[C], PR.chemsc[H])";
	 ERROR = "%2.2fppm" % "(PR.errppm)";
	NAME = "PG [%d:%d]" % "((PR.chemsc)[C] - 6, (PR.chemsc)[db] - 2.5)";
	SPECIE = "PG [%d:%d / %d:%d]" % "(FA1.chemsc[C], FA1.chemsc[db] - 1.5,
FA2.chemsc[C], FA2.chemsc[db] - 1.5)";
	PRECURINTENS = PR.intensity;
	FAS = sumIntensity(FA1, FA2);;


################ end script ##################



##########################################################
# Identify PI with checking  the precursor mass          #
##########################################################


QUERYNAME = Phosphatidylinositol;
DEFINE PR = 'C[37..53] H[30..140] O[13] P[1]' WITH DBR = (3.5,10.5), CHG = -1;
DEFINE headPI = 'C[6] H[10] O[8] P[1]' WITH DBR = (1.5,4.5), CHG = -1;
DEFINE FA1 ='C[14..22] H[20..50] O[2]' WITH DBR = (1.5,7.5), CHG = -1;
DEFINE FA2 ='C[14..22] H[20..50] O[2]' WITH DBR = (1.5,7.5), CHG = -1;
```

```
IDENTIFY Phosphatidylinositol WHERE
	# marking
	PR IN MS1- AND
	FA1 in MS2- AND
	FA2 in MS2- AND
	headPI in MS2-


SUCHTHAT
	FA1.chemsc + FA2.chemsc + headPI.chemsc + 'C3 H6 O1' == PR.chemsc


REPORT
	MASS = "%4.4f" % "(PR.mass)";
	CHEMSC = PR.chemsc;
	COMPSC = "PO%dC%dH%d" % "(PR.chemsc[O], PR.chemsc[C], PR.chemsc[H])";
	 ERROR = "%2.2fppm" % "(PR.errppm)";
	NAME = "PI [%d:%d]" % "((PR.chemsc)[C] - 9, (PR.chemsc)[db] - 3.5)";
	SPECIE = "PI [%d:%d / %d:%d]" % "(FA1.chemsc[C], FA1.chemsc[db] - 1.5,
FA2.chemsc[C], FA2.chemsc[db] - 1.5)";
	PRECURINTENS = PR.intensity;
	FAS = FA1.intensity + FA2.intensity;;


################ end script #################



##########################################################
# Identify PS with checking  the precursor mass, NLS and FAS          #
##########################################################


QUERYNAME = Phosphatidylserine;
DEFINE PR = 'C[34..50] H[30..120] O[10] N[1] P[1]' WITH DBR = (3.5,10.5), CHG = -1;
DEFINE headPS = 'C[3] H[5] O[2] N[1]' WITH DBR = (-0.5,6.5), CHG = 0;
DEFINE FA1 ='C[12..22] H[20..50] O[2]' WITH DBR = (1.5,7.5), CHG = -1;
DEFINE FA2 ='C[12..22] H[20..50] O[2]' WITH DBR = (1.5,7.5), CHG = -1;


IDENTIFY Phosphatidylserine WHERE
	# marking
	PR IN MS1- AND
	FA1 in MS2- AND
	FA2 in MS2- AND
	headPS in MS2-


SUCHTHAT
	FA1.chemsc + FA2.chemsc + headPS.nlsc + 'C3 H6 P1 O4' == PR.chemsc


REPORT
	MASS = "%4.4f" % "(PR.mass)";
	CHEMSC = PR.chemsc;
	COMPSC = "PO%dNC%dH%d" % "(PR.chemsc[O], PR.chemsc[C], PR.chemsc[H])";
	 ERROR = "%2.2fppm" % "(PR.errppm)";
	NAME = "PS [%d:%d]" % "((PR.chemsc)[C] - 6, (PR.chemsc)[db] - 3.5)";
	SPECIE = "PS [%d:%d / %d:%d]" % "(FA1.chemsc[C], FA1.chemsc[db] - 1.5,
FA2.chemsc[C], FA2.chemsc[db] - 1.5)";
	PRECURINTENS = PR.intensity;
	FAS = sumIntensity(FA1, FA2);;
```

```
################ end script ##################


##########################################################
# Identify PA with checking  the precursor mass and FAS         #
##########################################################


QUERYNAME = PhosphatidicAcid;
DEFINE PR = 'C[31..47] H[30..120] O[8] P[1]' WITH DBR = (2.5,9.5), CHG = -1;
DEFINE FA1 ='C[12..22] H[20..50] O[2]' WITH DBR = (1.5,7.5), CHG = -1;
DEFINE FA2 ='C[12..22] H[20..50] O[2]' WITH DBR = (1.5,7.5), CHG = -1;


IDENTIFY PhosphatidicAcid WHERE
       # marking
       PR IN MS1- AND
       FA1 in MS2- AND
       FA2 in MS2-


SUCHTHAT
       FA1.chemsc + FA2.chemsc + 'C3 H6 P1 O4' == PR.chemsc


REPORT
       MASS = "%4.4f" % "(PR.mass)";
       CHEMSC = PR.chemsc;
       COMPSC = "PO%dC%dH%d" % "(PR.chemsc[O], PR.chemsc[C], PR.chemsc[H])";
       ERROR = "%2.2fppm" % "(PR.errppm)";
       NAME = "PA [%d:%d]" % "((PR.chemsc)[C] - 3, (PR.chemsc)[db] - 2.5)";
       SPECIE = "PA [%d:%d / %d:%d]" % "(FA1.chemsc[C], FA1.chemsc[db] - 1.5,
FA2.chemsc[C], FA2.chemsc[db] - 1.5)";
       PRECURINTENS = PR.intensity;
       FAS = sumIntensity(FA1, FA2);;

################ end script ##################
```
